# Communication in Neglected Tropical Diseases’ elimination: A scoping review and call for action

**DOI:** 10.1371/journal.pntd.0009774

**Published:** 2022-10-13

**Authors:** Claudia Nieto-Sanchez, David M. Hatley, Mario J. Grijalva, Koen Peeters Grietens, Benjamin R. Bates

**Affiliations:** 1 Socio-Ecological Health Research Unit, Department of Public Health, Institute of Tropical Medicine, Antwerp, Belgium; 2 Department of Epidemiology, University of London School of Hygiene & Tropical Medicine, London, United Kingdom; 3 Infectious and Tropical Disease Institute (ITDI), Department of Biomedical Sciences, Heritage College of Osteopathic Medicine, Ohio University, Athens, Ohio, United States of America; 4 Center for Research in Health in Latin America (CISeAL), Facultad de Ciencias Exactas y Naturales, Pontifical Catholic University of Ecuador, Quito, Ecuador; 5 Nagasaki, School of Tropical Medicine and Global Health, Nagasaki University, Nagasaki, Japan; 6 School of Communication Studies, Ohio University, Athens, Ohio, United States of America; Princeton University, UNITED STATES

## Abstract

**Background:**

Although the practice of communication is often called upon when intervening and involving communities affected by NTDs, the disciplinary framework of health communication research has been largely absent from NTD strategies. To illustrate how practices conceptualized and developed within the communication field have been applied in the context of NTD elimination, we conducted a scoping review focusing on two diseases currently targeted for elimination by the WHO: lymphatic filariasis and Chagas disease.

**Methods:**

We examined studies published between 2012 and 2020 in five electronic databases. Selected articles were required to (i) have explicit references to communication in either the abstract, title, or key words; (ii) further elaborate on the search terms (communication, message, media, participation and health education) in the body of the article; and (iii) sufficiently describe communication actions associated to those terms. Using the C-Change Socio-Ecological Model for Social and Behavior Change Communication as a reference, the articles were analysed to identify communication activities, theoretical frameworks, and/or rationales involved in their design, as well as their intended level of influence (individual, interpersonal, community, or enabling environment).

**Results and implications:**

A total of 43 articles were analysed. Most interventions conceptualized communication as a set of support tools or supplemental activities delivering information and amplifying pre-defined messages aimed at increasing knowledge, encouraging community involvement, promoting individual behavior change, or securing some degree of acceptability of proposed strategies. Although important attempts at further exploring communication capabilities were identified, particularly in participation-based strategies, for most studies, communication consisted of an underdeveloped and under-theorized approach. We contend that a more complex understanding of the capacities offered by the health communication field could help attain the biomedical and social justice goals proposed in NTD elimination strategies. Three ways in which the field of health communication could further enhance NTD efforts are presented: informing interventions with theory-based frameworks, exploring the political complexity of community participation in specific contexts, and identifying conceptualizations of culture implied in interventions’ design.

**Conclusion:**

This article is a call to action to consider the resources offered by the health communication field when researching, designing, or implementing NTD interventions.

## Author summary

Communication is often seen as a central tool in NTD elimination strategies. However, the disciplinary framework of communication research is rarely considered in interventions’ design. To understand how communication action is conceived and practiced, we conducted a scoping review with a focus on two diseases currently targeted for elimination: lymphatic filariasis and Chagas disease. We examined 43 studies published between 2012 and 2020 to identify communication practices, theoretical frameworks informing communication action and levels of intended influence. Community participation was the goal most commonly pursued through communication action, both in LF and CD strategies; important attempts at expanding the scope and reach of this practice were identified. However, our results indicate that while the field of health communication has gained complexity in the understanding of the nature of communication ecologies in public health interventions, NTD research has maintained a superficial involvement with health communication theory and science. This article is a call to action to consider the resources offered by the health communication field when researching, designing, or implementing NTD interventions to enhance the possibilities of communication action in implementation settings.

## Introduction

In 2005 the World Health Organization (WHO) introduced the category of neglected tropical diseases (NTDs) to make the impact of tropical diseases on populations living in poverty a global priority [1]. From the onset, neglect was characterized as a complex problem involving multiple actors and interacting at three different scales:

At the community level, fear and stigma can sometimes lead sufferers and their families to conceal their condition. At the national level, these diseases are often hidden–out of sight, poorly documented, and silent, as those most affected have little political voice. As a result, neglected diseases are rarely given high priority by ministries of health or finance in endemic countries. Neglected diseases lack visibility at the international level as well. Tied as they are to specific geographical and environmental conditions, they are not perceived as direct threats to industrialized countries. [2] (p. 6)

The WHO’s NTD strategy has obtained financial and political support and high visibility through their inclusion in the Sustainable Development Goals in 2015 [3,4] and the inauguration of the World Neglected Tropical Diseases Day on January 20, 2020 [5]. The NTD acronym is now a well-established term [6]. By these measures, the WHO’s strategy is a success.

Although the NTD strategy emphasizes poverty alleviation [7], the WHO’s strategy has been criticized for avoiding debates about the political, economic, and social systems that increase vulnerability [8]. In response, social scientists have been employed to understand the blind spots of the NTD strategy at multiple levels, from the heterogeneous impacts of NTD on individual demographic factors [9–11], to larger forms of vulnerability linked to precarity of living environments and working conditions [12–14]. Social and environmental factors have been identified as important drivers of disease [15,16], while stigma deters populations’ access to treatment and reflects larger forms of exclusion [17–19]. To address these concerns, anthropologists, sociologists and economists have been invited to assist in NTD research and implementation [20–24]. Although “communication” is often claimed to be an easy and cost-effective tool for addressing NTDs, health communication research as a disciplinary framework, and the communication theories that drive it, have been largely absent from this discussion. In addition, practices described under the ‘communication’ label are broad and diverse, making it difficult to understand the actual contribution that communication action can make.

To illustrate how communication has been conceptualized and applied in the context of NTD elimination, we conducted a scoping review focusing on two diseases: lymphatic filariasis (LF) and Chagas disease (CD). Although current elimination efforts for both CD and LF rely heavily on prevention [25], their elimination strategies significantly differ. LF’s elimination strategy is aimed at stopping transmission, mainly through community wide administration of anti-parasitic drugs for a minimum of five years in endemic areas [26]. Control methods for CD, on the other hand, are focused on interrupting the transmission cycle between vectors and humans through selective or community wide indoor spraying with insecticide (deltamethrin), accompanied by information and education activities [27,28]. Environmental modifications through improvement of housing infrastructure have also been implemented in areas with presence of endemic species of triatomines (e.g. *T*. *dimidiata* in Central America and *R*. *ecuadoriensis* in Ecuador) to extend the protective capacity of other measures [29,30].

This scoping review aims to describe how communication practices in LF and CD interventions have been put into effect by analyzing published literature on both diseases from a health communication point of view. By synthesizing existing information, this review aims to, first, establish how NTD prevention communicators currently understand what “communication” is. Then, we seek to expand this relatively limited understanding by showing how the broader sense of communication theories, methods, and actions offered by the discipline of health communication can support current NTD efforts.

### Health communication: From persuasion to dialogue

Covering the full extent and evolution of the health communication field is beyond the purposes of this manuscript. However, it is important to situate health communication as a discipline with distinct theories and research methods. If communication is the fundamental human mode of explaining how we, as humans, relate to the world we live in [31], health communication studies how humans explain their experience of health and disease. Because of its symbolic character, communication lies at the core of the definition of physical states as characterized in medical, cultural, spiritual, emotional, and ethical systems. Also, as a metalanguage, communication processes and resources allow human beings to name the world and their embodied experiences, social relationships, and ideological expectations [32]. Under those premises, health communication theories are frameworks purposefully elaborated to explain and influence communication practices in relation to health and illness [33].

During the post-World War II period, health communication was conceived as a mere tool to facilitate the delivery of health products and services [34]. The underlying assumption was that the evaluation of good or ill health had to rely on specialized scientific knowledge held by experts. As such, communication played the supporting role of ensuring the clear transmission of technical information from experts to the public. Media theories popular in the 1950s and 60s endorsed this perspective under the assumption that health issues could be prevented if people were sufficiently informed and educated about the risks they faced. Concurrently, psychological theories provided explanations about cognitive functions underlying individual mental processes that informed persuasive campaigns and messages designed to influence behavior [35].

The field expanded in the 1970s and 80s through the development of socio-cultural perspectives that questioned the utilitarian nature of traditional information transmission. In these socio-cultural perspectives, communication is not simply a speaker sending a message to a receiver; rather, communication frames the meaning of health in relation to who participates in its definition, under what role, and through which languages [36]. Consequently, knowledge of the world is produced, not simply transmitted and apprehended [37]. With this turn, beginning in the 1970s, health communication practitioners shifted their attention towards factors that influence practices aimed at obtaining information, applying preventive practices, measuring risk, deciding about treatment options, and dealing with disease and death. The premise behind this conceptual move was that, after a communicator identifies these factors, the communicator can more strategically design persuasive efforts.

Both utilitarian and socio-cultural considerations of health communication have been extensively used to pattern governmental, commercial, and non-profit communication efforts aimed at persuading audiences into pre-defined behaviors and actions. Because of their emphasis on knowledge and decision making, these strategies are closely linked to health literacy, health education, and health promotion in public health [38].

In the 1990s and 2000s, a turn to community participation has become a central issue in the evolution of the field of communication. People can be co-creators of communication processes and not just uniform recipients of information and messages. For the last few decades, communication and development specialists who emphasize participatory approaches to communication have argued that top-down models to promote behavior change are not sustainable and that there is an ethical obligation to enhance the agency of marginalized populations through communication interventions [39,40]. Communication for social change, advocacy and social mobilization models have flourished as part of this turn. Participatory decision-making has been extensively studied in the literature of social development [41], and re-emphasized by policies, plans, and interventions in virtually every single area of social development. In spite of this resonance, the term “participation” has been increasingly problematized by researchers and program designers challenged by the multiple meanings assigned to this concept, and as a consequence, difficulties in measuring its impact [42–45].

More recent approaches have proposed dialogic models as a mode of questioning power relations traditionally existing in research contexts where strategic communication actions are designed [46,47]. Arthur Frank describes dialogical research as an encounter in which both the researcher and participants are subjects of change [48]. As such, instead of assuming the position of a detached external observer, the dialogical researcher admits that communication could be misleading and ineffective if it is focused on decontextualized persuasion or does not seek to learn from the audience that it addresses. Greiner [49] expands on this idea by emphasizing the communicative character of the conceptualization, design and delivery of interventions. Interventions can be organized as possibilities offered to a community of deliberating individuals grappling with valid, complex, and dynamic elements influencing their individual and social life. In this perspective, communicators should not create a set of messages developed outside the community and strategically articulate them to persuade masses of people towards predefined interventions. This call for health intervention professionals to engage community members in authentic deliberation over goals, worldviews, and methodologies for attaining those goals–in short, a call for a dialogic perspective–has been strongly promoted in decolonizing debates in the last few years [50].

Although there has been a great deal of conceptual development in the field of health communication to expand what is meant by “communication,” it is not known how extensively practitioners in NTD contexts have adopted the turn from top-down transmission of information to participatory co-creation of meaning. That is, we do not know how extensively NTD communicators have adopted the theories, practices, and levels of change encouraged by contemporary health communication theory. To assess this, we performed a scoping review.

## Methods

### Data sources

We performed a scoping review [51] following the PRISMA Extension for Scoping Reviews (PRISMA-ScR) guidelines (see S1 Table). This scoping review identified studies published between January 2012 and March 2020, that implemented communication activities as part of LF or CD elimination strategies. This timeframe covers the period from the launch of the London Declaration in June 2012 to the declaration of the Covid-19 pandemic. The London Declaration was chosen as the starting point because it represented a significant financial commitment to control, eliminate or eradicate ten NTDs by 2020, endorsed by pharmaceutical companies, donors, endemic countries, donor countries, and non-government organizations. The onset of the Covid-19 pandemic was chosen as the stopping point under the assumption that NTD programing and publications would be affected by public emphasis on this newly emergent disease.

Full searches were conducted using five electronic databases: Medline-Pubmed, CINAHL, PsycINFO, LILACS, and Web of Science. To identify the most common terms associated with communication practices, an initial search was carried out using the word ‘communication’ as the only search term. Based on the results obtained, four more terms were used to redefine the search strategy: ‘message’, ‘media’, ‘participation’, and ‘health education’. Expressed in Boolean terms, the general search strategy was: “(lymphatic filariasis OR Chagas disease) AND (communication OR message OR media OR participation OR health education).” Although participation and health education could be considered distinctive endeavors, interventions usually include some form of communication action under such labels. A decision was made to include “participation” and “health education” as key terms to increase accuracy of this review.

Articles were selected in two phases. During the first phase, titles, abstracts, and key words of all the identified studies were examined. After the elimination of duplicates (i.e., the second, third, or fourth instance of each article that appeared in multiple databases), abstracts were reviewed to determine fitting of search terms to the objectives of this scoping review. Three exclusion criteria were considered at this stage: (1) use of search terms for purposes different from communication action, including communication as biological interaction (e.g., cell-cell communication, pheromone production, chemical signals between plants and animals); media as an environmental condition in laboratory settings or publication outlet; and participation as interaction in biological processes; (ii) impossibility to assess the full manuscript due to language constraints (only manuscripts written in English and Spanish were retained); and (iii) type of manuscript (short communications, opinion pieces, editorials to special numbers, theses, news reports and conferences’ presentations were excluded). During the second phase, author CN-S and BB screened the full text of the retained manuscripts and used two additional criteria to determine their eligibility for this review. Only articles that (i) further elaborated on the search terms in the body of the article and (ii) sufficiently described communication activities associated to those terms were finally considered for further analysis. Citations and abstracts were then downloaded into Mendeley reference manager.

### Data extraction and synthesis

**Fig 1:**
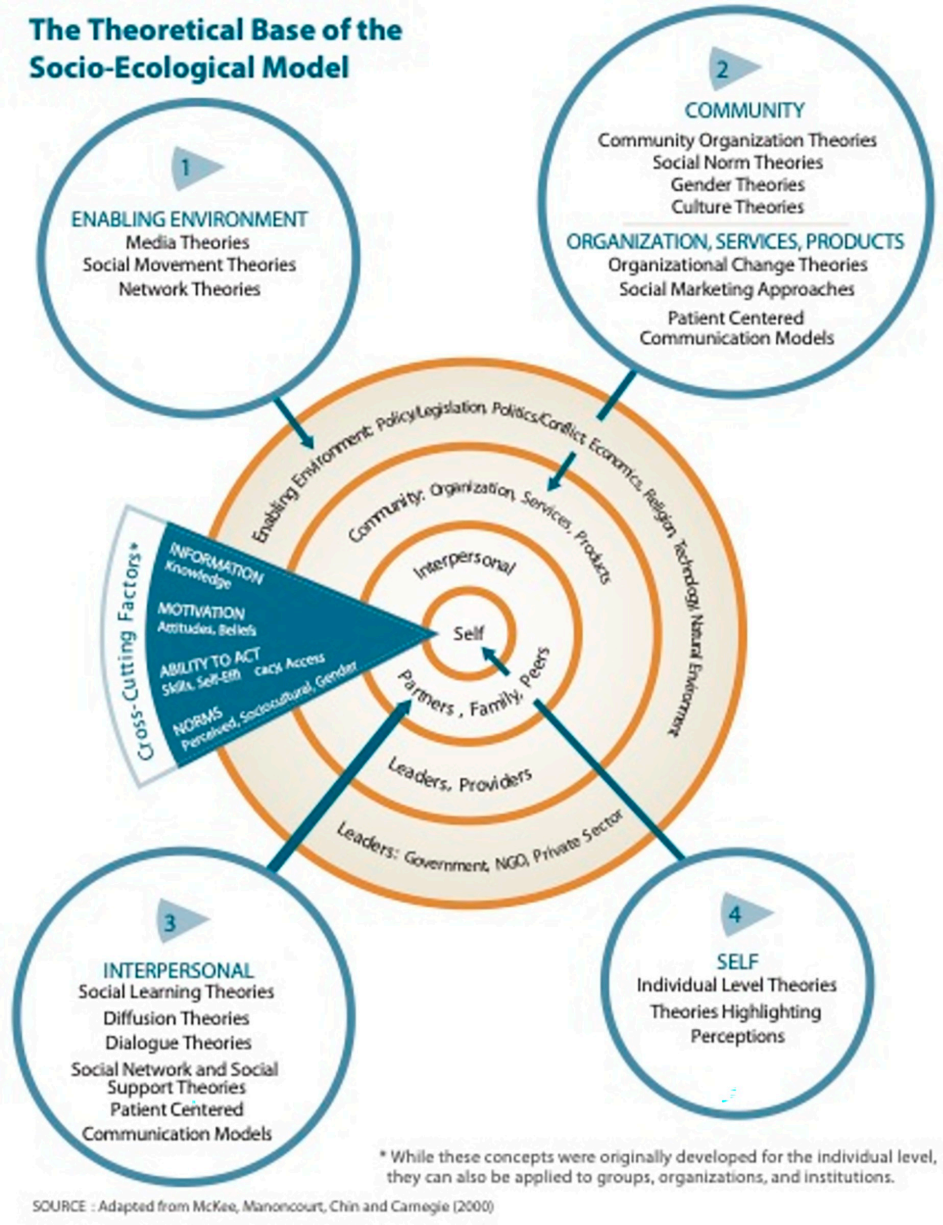
The Theoretical Base of the Socio-Ecological Model C-Change. Use authorized by FHI 360. Original source: *C-Change*. *2012*. *C-Modules*: *A Learning Package for Social and Behavior Change Communication (SBCC)*. *Washington*, *DC*: *C-Change/FHI 360*.

The C-Change Socio-Ecological Model for Social and Behavior Change Communication was used as the main analytical framework for this review [52] (Fig 1). This framework was initially developed by USAID, as part of the Communication for Change project (C-Change). The *C-Change* project worked to develop evidence-based learning tools to aid organizations interested in implementing communication strategies to support health and development interventions. The *C-Change* framework was selected for two reasons. First, it argues that communication is most successfully when it is theoretically informed. Although the *C-Change* model was designed to inform social and behavior change with an emphasis on communication theory, its theoretical base also includes a wide range of concepts derived from fields like psychology, political sciences and anthropology that support strong communication practices. Instead of seeing theories from a disciplinary perspective, *the C-Change model* considers the practical applications of theory in communication practice.

Second, the *C-Change* framework reflects the fact that communication interventions are embedded in larger systems. Accordingly, it synthesizes and organizes communication theories according to their capacity to influence practices at individual, interpersonal, community, and environmental levels, which can be an important factor in decision making around communication activities in NTD interventions.

Data was extracted by author CN-S using a Word extraction form for each focalized NTD (LF and CD). The following data points were organized in the extraction tables: authors, year of publication, country of intervention, stated rationale for developing communication action, references to particular theoretical frameworks, and implemented communication actions as described in each manuscript (S2 and S3 Tables). Using the *C-Change* framework, communication activities were coded according to their main levels of intended influence (individual, interpersonal, community, or enabling environment), and intended impacts (knowledge, perception, community participation, etc.). The wording used by the author of each article was extracted for analysis of communication actions.

## Results

**Fig 2:**
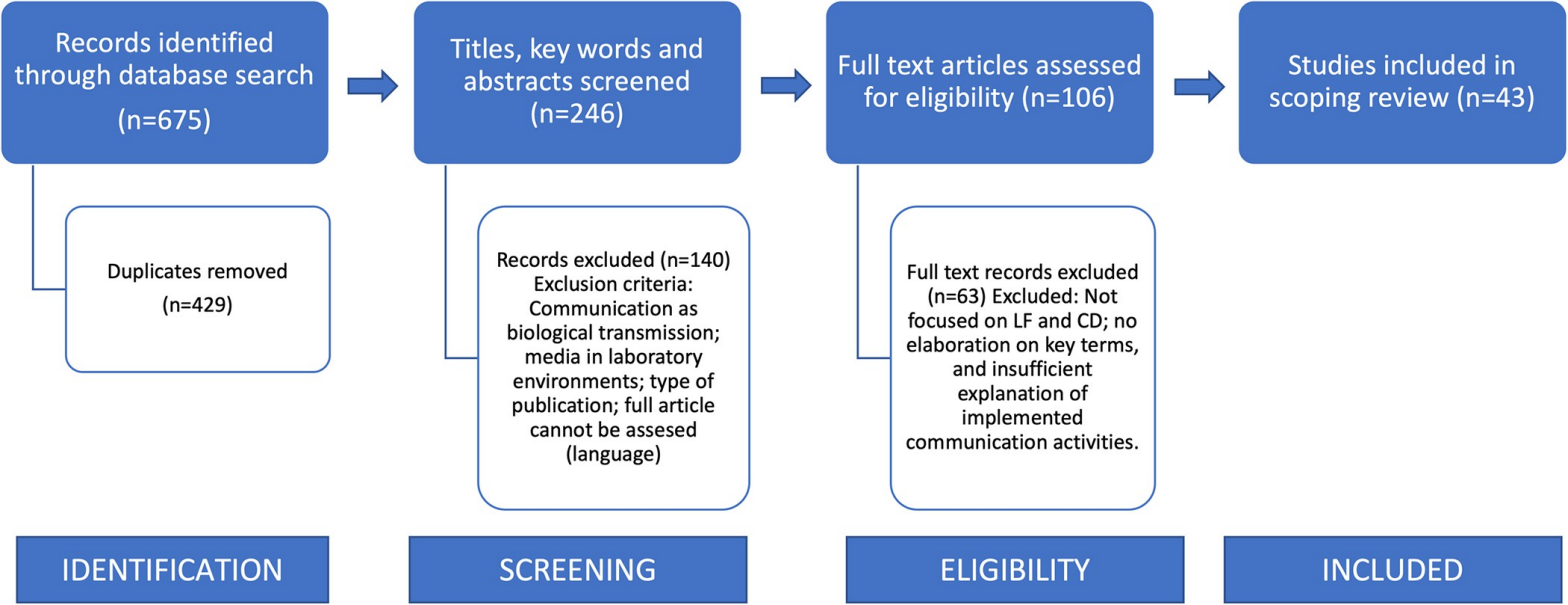
Flow chart of records’ selection.

A total of 675 articles were identified during the first phase of data screening. After the elimination of duplicated articles (n = 429), 246 abstracts were reviewed for relevance to the purposes of this review. After this selection, 106 manuscripts were retained for the second phase of screening and 43 of them, 18 of which were focused on LF and 25 on CD, were finally considered for analysis (Fig 2).

A summary of the results can be found in Tables 1 and 2. Manuscripts and excerpts included in this analysis are presented as supplementary materials (S2 and S3 Tables). We now present the communication theories employed, the level of change communicative activities targeted, and the communication activities employed for each disease.

**Table 1:** Simplified summary of findings of communication approaches in lymphatic filariasis’ manuscripts (n = 18).

Aspects assessed	N	%
**Theory used**		
No theory	14	77.7%
Information, Education & Communication	3	16.7%
Community Based Participatory Research	1	5.6%
**Level of change**		
*Individual*	*9*	*50%*
Knowledge	7	38.9%
Perception	1	5.6%
Experience	1	5.6%
Behavior change	1	5.6%
*Interpersonal*	*3*	*16*.*7%*
Diffusion	2	11%
Social networks	1	5.6%
*Community*	*6*	*33*.*3%*
Participation	5	27.8%
Collective action	2	11%
Structure of programs and services	1	5.6%
*Enabling environment*	-	-
**Communication activities**		
*Transmission* (top-down)		
Health education (community awareness, sensitization)	8	44.4%
Message design / dissemination	7	38.9%
Communication materials (distribution)	5	27.8%
Workshops/meetings	3	16.7%
Behaviors’ demonstration	2	11%
*Dialogic* (feedback loops)		
Insertion in existing networks/social structures	5	27.8%
Community partnerships	3	16.7%
Trust building	2	11%
Interactive education activities	1	5.6%

* Totals and percentages within level of change and communication do not total to 18 to account for multiple levels/activities reported in an article. See S2 Table for additional information.

**Table 2:** Simplified summary of findings of communication approaches in Chagas disease’s manuscripts (n = 25).

Aspects assessed	N	%
**Theory used**		
No theory	14	56.0%
EcoHealth Model (participation)	4	16.0%
PRECEDE-PROCEED model	2	8.0%
Participatory Action Research	2	8.0%
Diffusion of Innovations Model	1	4.0%
Behavioral Design	1	4.0%
Health Belief Model	1	4.0%
**Level of change**		
*Individual*	*6*	*24%*
Knowledge	5	20%
Risk perception / vulnerability	1	4%
*Interpersonal*	*1*	*4%*
Diffusion	1	4%
*Community*	*19*	*76%*
Participation	11	44%
Structure of programs and services	4	16%
Ownership	2	8%
Patient-centered communication	1	4%
Meaning making	1	4%
Social marketing	1	4%
*Enabling environment*	*2*	*8%*
Media	1	4%
Coalition building	1	4%
**Communication activities**		
*Transmission (Top-Down)*		
Workshops/meetings/house visits	10	40%
Communication materials (distribution)	8	32%
Health education (community awareness, sensitization)	7	28%
Message design / dissemination	3	12%
*Dialogic* (Feedback loops)		
Community partnerships	6	24%
Integration of local knowledge	6	24%
Insertion in existing networks/social structures	4	16%
Interactive education activities	2	8%

* Totals and percentages within level of change and communication do not total to 25 to account for multiple levels/activities reported in an article. See S3 Table for additional information.

### Communication actions in lymphatic filariasis (LF) elimination

#### Theories employed

More than three-quarters (n = 14, 77.7%) of communication interventions to address LF did not use a theoretical framework. In the remainder, two theoretical frameworks from communication theory were used in LF interventions. Information, education and communication (IEC) theory was used in 3 studies (16.7%) [53–55]. Community-based participatory approaches were used in the remaining study (5.6%) [56].

*Level of change*. The interpersonal level of change was most likely to be addressed in LF interventions, followed by the community and interpersonal levels, respectively. Half of the studies targeted the individual level of change (n = 9, 50%). At this individual level, studies focused on increasing knowledge (n = 7) [53,54,57–61], identifying perceptions (n = 1, 5.6%) [62], capturing experiences (n = 1, 5.6%) [61], and promoting behavior change (n = 1, 5.6%) [55]. About one-third of the studies addressed the community level of change (n = 6, 33.3%). These studies called for increasing community participation (n = 5, 27.8%) [55,56,63–65], facilitating collective action (n = 2, 11%) [64, 66], or altering the structure of programs and services (n = 1, 5.6%) [63]. About one in eight studies targeted the interpersonal level of change (n = 3, 16.7%). Interventions focused on the interpersonal level emphasized disseminating information (n = 2, 11.0%) [67,68] and using social networks (n = 1, 5.6%) [69]. No study addressed the enabling environment as a level for change.

#### Communication activities performed

The studies focused on LF elimination performed communication activities under a top-down approach in which the goal was to communicate information from health actors to the population. Dialogic activities were performed less often. As indicated in the simplified table of results (Table 1), health education strategies were the most common approach (n = 8, 44.4%) [53–55,58–60,62,67], followed by message design and dissemination (n = 7, 33.3%) [53,58–61,64,69], distribution of communication materials (n = 5, 27.8%) [53,54,59,67,68], and insertion of messages into existing networks and social structures (n = 5, 27.8%) [63–65,69,70]. Significantly, only a couple of cases used these four common strategies under a dialogic angle [64,70]. Because the communication activities employed were diverse, it is important to briefly delineate the specific activities performed in these studies to reflect the array of approaches taken.

Top-down communication activities included activities designed to persuade asymptomatic populations to take medicine [57], ensure coverage [62], and mobilize local populations to take part in MDA programs [63,68,71]. Health literacy interventions were also used to address issues of low coverage [56,57,59]. LF-specific communication activities were also conceived as means to educate general and at-risk populations [58], and raise awareness to move forward elimination goals [59,60]. Emphasis is placed on timely provision of information regarding access to treatment making positive results visible [61]. Because transmission of information was considered the most significant component to promote preventive action, several studies employed health education activities to facilitate involvement of health personnel and local communities [57,58,60]. Workshops and demonstrations to show care practices to community members supplemented health education [67,72].

In addition to didactive activities, several LF studies used sensitization strategies, or strategies that raise awareness and acceptance of a health intervention. As another top-down approach, sensitization activities provided information to health personnel so it could flow downward to communities and individuals [57,68]. Involving health workers or health volunteers [58], community drug distributors [55], health activists [54], nurses, school teachers, or political leaders [53], as well as working with existing local structures such as village health forums [65], schools and churches [53], were also proposed as effective strategies to disseminate information and deliver interventions. Even the family is seen as a persuasive channel, for shaping treatment priorities [53,54,67]. As part of this campaigns to raise awareness and acceptance, traditional media such as radio and newspapers, as well as local forms of message dissemination (such as speakers installed in vehicles [53]), were used. In these sensitization strategies, several studies sought to adapt messages to the local population’s culture [64], and community dynamics and structural factors to promote MDA [55,56].

A smaller, but significant, set of dialogic communication activities were also employed. Top-down activities typically dismiss the political and cultural relevance of communities’ opinions, beliefs and perceptions [64,66,73]. As such, dialogic communicators claim that top-down LF strategies too often neglect contextual, economic, and political factors [63,64]. Dialogic approaches, such as community partnership seek to directly incorporate patients’ experience [61], existing local social structures [65], and respect for local knowledge [64] [66] in implementation efforts. In these approaches, interpersonal communication is used to facilitate trust-based relationships rather than as a channel to send a top-down message [68,69]. Similarly, campaign resources are not used to develop slicker and more sophisticated messages but, instead, address community members’ legitimate concerns [73].

### Communication actions in Chagas disease (CD) control/ elimination efforts

#### Theories employed

More than half (n = 14; 56.0%) of the communication interventions to address CD did not use a theoretical framework. Use of specific theoretical frameworks in the design of communication interventions and activities was, however, more frequent in CD than in LF interventions. In the remaining studies, six different theoretical frameworks were employed. The most common model was the EcoHealth Model (n = 4, 16.0%) [74–77], followed by Participatory Action Research (n = 2, 8.0%) [77,78] and the PRECEDE-PROCEED model (n = 2, 8.0%) [77,79]. The Health Belief Model [80], Diffusion of Innovations theory [76], and Behavioral Design [81] were used in 1 study each.

#### Level of change

The community level of change was the most likely to be addressed in CD elimination, followed by the individual level, the enabling environment level, and the interpersonal level. A preponderance of the studies sought change at the community level (n = 19, 76.0%). Interventions at the community level were aimed at promoting community participation (n = 11, 44%) [74–78,80–85], altering the structure of programs and services (n = 4, 16%) [79,86–88], promoting ownership (n = 2, 8%) [77,89], exploring meaning making (n = 1, 4%) [90], testing patient-centered approaches (n = 1, 4%) [91], and contributing to community-based social marketing strategies (n = 1, 4%) [92]. Six studies targeted the individual level of change (n = 6, 24%). These interventions were directed towards increasing individual knowledge (n = 5, 20%) [80,93–96] or describing perceptions of risk (n = 1, 4%) [97]. Two studies (8%) sought change at the level of enabling environments. These two studies sought to involve the construction and agriculture sectors to produce more stable incomes that would increase community resistance to CD [77,86]. Significantly, some studies sought change at multiple levels, as their interventions included multidisciplinary [75], multi-component [92] or multisectorial methodological designs [85,95].

#### Communication activities performed

Most studies on CD elimination performed communication activities as top-down activities. As compared to LF elimination however, CD elimination used dialogic approaches more often. As indicated in the simplified table of results (Table 2), several of the same top-down communication strategies were used for CD as for LF, including: health education strategies (n = 7, 28%) [77,84,86,88,89,94,96], message design and dissemination (n = 3, 12%) [82,87,91], and distribution of communication materials (n = 8, 32%) [78,82,84,92,95,96,98,99]. Unlike for LF, top-down communication activities for CD also used workshops and meetings as a very common strategy (n = 10, 40%) [75,76,79,82,83,85,89,92,94,100]. The most common dialogic strategies for CD was integration of local knowledge into messaging (n = 6, 24%) [75,82,83,89,94,100], as well as forming community partnerships (n = 6, 24%) [76–78,81,82,92], followed by insertion of messages into existing networks and social structures (n = 4, 16%) [87,88,95,100]. Significantly, only one of these four common strategies was dialogic.

As with the communication activities for LF elimination, CD elimination efforts employed a wide array of strategies. Therefore, it may be useful to briefly illustrate specific activities performed in these studies to reflect the diversity of approaches taken.

Top-down approaches at the community level focused on the interactions between health personnel and local communities. These strategies included enrolling community members in epidemiological and entomological surveillance [79,81,82,84,92]. Health promoters were encouraged to provide information about detecting, collecting, and reporting triatomines community members [79,84,88,101]. Other studies noted that a lack of trust in health authorities limited the persuasiveness of messages and sought to influence that factor [87].

Several studies sought to raise awareness of CD through health education. These efforts assume that understanding the transmission cycle for CD is a fundamental condition to enhance acceptability and effectiveness of prevention measures [74,98]. Didactic education was used to explain the CD transmission cycle and spread information about home-based preventive practices through classroom lessons [89,92,102], workshops [94], contests [97] and public activities in community settings [95]. Education-based strategies also distributed information to the community through booklets [84,101,103], video and radio resources [90,98], and mobile telephones [91], as well as other materials such as posters, printed bags, magnets, and lottery tickets with key messaging used for community outreach [78,90,92,95].

Dialogic approaches to communication for CD elimination employed a variety of participatory planning and implementation processes [75,79,88]. Approaching local populations to collect traditional knowledge [74,78], involving local forms of organization [81], and gaining insights into the experience of patients and affected communities to formulate interventions [85,90,95] were attempts at bringing more complex participatory strategies into design and implementation efforts. In particular, a focus on women [76] and marginalized communities as key partners in dialogue was encouraged [87]. This local knowledge was also incorporated into citizen science as a health literacy strategy [93,102].

The most extensive use of dialogic approaches was identified in interventions that employed the Ecohealth perspective. While considering the impact of built and natural environments on disease transmission, Ecohealth approaches in the case of CD also explored participatory approaches to facilitate collaborations. Ecohealth at the household level, for example, emphasized collaboration among family members for home construction or reconstruction, waste management, and animal rearing practices [74–77]. Authors using the Ecohealth perspective also emphasized the need for long-term health promotion integrated into local health systems to extend application of protective practices over time [74,83]. Advocates of this, and other dialogic approaches, also argued that, when compared to top-down approaches, dialogic approaches were associated with increased knowledge of CD and adoption of home-based triatomine control practices [77], as well as greater sustainability [86], cost-effectiveness [94], and improved health outcomes [82].

## Discussion

The results of this review indicate that communication in LF and CD interventions has been largely used as a set of support tools and supplemental activities aimed at achieving biomedical objectives. Most of the use of communication follows a traditional, top-down and linear conception of communication. Although activities varied in channels and approaches, communication actions were mostly aimed at delivering information and amplifying pre-defined messages to increase knowledge and participation, promote individual behavior change, or securing some degree of acceptability for proposed control and prevention strategies.

Although these trends were quite consistent in most of the manuscripts, important attempts at further exploring communication capabilities were also identified. For example, CD researchers attempted complex forms of community involvement, partially in response to historically established close association between living environments and CD. This complexity also meant that CD researchers were more likely to use theoretical perspectives to design their communication activities. CD researchers were also more likely to include activities generating critical thinking and knowledge exchange between scientists and communities. Similarly, LF researchers showed the limitations of associating community participation with adherence to treatment in MDA strategies. Those limitations were expressed as a critique of the apolitical approach that most community participation strategies take, despite the fundamental role that highly political concepts such as decision-making, representation and autonomy play in these cases.

Results also indicate that NTD research has maintained a superficial involvement with health communication theory and science. That is, although the field of health communication offers a rich body of communication theory and methodology that could be used for NTD research, NTD research has not taken full advantage of this resource. For most studies, communication consisted of an underdeveloped and under-theorized approach, failing to take full advantage of the possibilities for effective communication to and with impacted individuals, communities, service systems, and sociopolitical environments. We contend that a more complex understanding of the processes and capacities offered by the health communication field could help better attain the medical and social justice goals proposed in elimination strategies. We present three ways in which the field of health communication could further enhance NTD efforts.

### Theory informed interventions

The complexity of factors involved in NTD occurrence demands a more specialized engagement with the theoretical frameworks supporting specific communication strategies. The lack of theory (and evidence based) decision-making around communication activities in interventions’ design is problematic. Untheorized implementation of communication strategies and activities under decontextualized assumptions can lead to resource underutilization and poor evaluation. In this review, we identified frequent misalignment between the rationales used to introduce communication actions and the actions implemented in consequence. Some interventions used theoretical frameworks designed to influence individual behavior but developed actions focused on community responses (in a misinterpretation of community participation as the result of adding up individual behaviors); in other cases, activities focused on one particular construct of a model (perceived risk), but interventions reported on communication aspects to be covered by the full model. As an example, our results indicate that persuasion towards behavior change is one of the outcomes most often expected from communication activities. However, persuasion is a difficult goal to attain as explained by the complexity of cultural, epidemiological, economic, technical and environmental arguments exposed by local communities to (fully or partially) accept or reject interventions (many of them reported in the reviewed articles). While individuals’ acceptance of MDA is often proposed as a logical aim to pursue under high coverage ambitions, strategies often ignore the fact that individual behavior is embedded in multiple social structures influencing individuals’ ability or even willingness to perform such behaviors. From the designers’ point of view, it would probably be more efficient to acknowledge that the MDA strategy has been conceptualized under a series of challenging premises (i.e., repeated medicine intake by asymptomatic individuals in contexts characterized by mistrust in Western medicine) that demand even more specific communication designs in which theoretical input could be useful. Dismissing refusers’ arguments as irrational and opting for persuasion as the fastest route to move forward elimination goals will only limit interventions’ response capacity in the long run [64].

In addition, we found that theoretical references were mostly used as explanatory models in analytical stages, but rarely considered for design or evaluation purposes. In the articles included in this review it was common to introduce a framework, but never report on the particular contributions or limitations of such framing to the results or the intervention itself. Oversimplification or overestimation of the actual capacity of specific communication strategies in and of themselves, as well as in support of elimination strategies, is to be expected from the atheoretical framing and assessment of interventions. The C-change model provides a basic entry point to further explore the theoretical underpinning of concepts and strategies commonly used in the communication filed, but of course, more developed resources are available.

### Complexities of participation processes

Although the idea of community participation has become commonplace in NTD literature, its implications for intervention design and implementation remain a challenge. In this review, participation was the communication action most commonly pursued. Activities conducted under this framing covered a wide range of schemes, from organization of sensitization meetings and provision of door-to-door information, to complex strategies requesting different forms of engagement from a wide range of actors. Actions designed to engage local stakeholders were included in this review under labels such as community participation, community engagement, social mobilization, sensitization, and health promotion. Manuscripts included in this review recommended extending the reach and scope of community participation, including engaging community members in the planning phase of interventions, relying on community knowledge for interventions design, and using locally relevant scenarios to enhance decision-making at community levels. However, each one of these approaches stems from different conceptual lenses and theoretical frameworks. Some authors supported the need to acknowledge the evolving character of establishing relationships with community members as a fundamental element in the generation of ownership and sustainability for health promotion efforts [64,66]. In other cases, deploying interventions through multiple channels and identifying diverse entry points to community scenarios was recommended to enhance engagement. Following this line of thought, interventions can go beyond populations’ buy-in by focusing on communication processes rather than outputs and facilitating flexible designs that can adapt to local realities and contextualize programmatic demands. As stated by Macharia et al., “Anticipating community participation in a programme is not an intelligent guess, as this is a learning process for beneficiaries and the stakeholders which can be earned through the sharing of experiences by all the concerned actors”[104]. Lines of research such as communication for development, communication for social change and dialogic communication can provide vast references on the complexity of generating participatory processes that consider power differentials, as well as ideas on how to incorporate the political aspect of participation into programs design. The case of polio vaccination can further illustrate this point [40,105].

### Culture-sensitive vs. culture-centered approaches

Overall, our results indicate that manuscripts rarely report on the local cultural frameworks operating in implementation settings. When these communication efforts engaged socio-cultural issues, they addressed culture either as a barrier or as an opportunity to help empower a population toward change. In health communication theory and practice, two parallel approaches have focused their attention on the interactions between community members and health personnel in multicultural health promotion efforts: the culture-sensitive and culture-centered approaches [106,107]. Although these two approaches acknowledge the relevant role of culture when setting specific health objectives, they hold distinct assumptions about the role culture plays in the consolidation of a healthy life. From a culture-sensitive perspective, culture is often viewed as a barrier to achieving “desired” health-outcomes; consequently, studies focus on identifying these barriers to frame health behaviors in a way that is acceptable for the local cultural landscape (i.e., system of beliefs, social and material relationships). The end-goal of culture-sensitive approaches is to bring into action preconceived health behaviors (and outcomes) by turning existing cultural features from barriers into facilitators [108]. Culture-centered approaches, on the other hand, see culture and local capabilities as the contextual factors that should define the logics of health interventions. Instead of barriers, they constitute a space to confront the dynamics of marginalization in which communities are situated [109]. Although most publications included in this review did not explore cultural factors in depth, the ones that did it operated somewhere between these two approaches. For interventions supporting culture sensitive approaches, sensitization and persuasion were commonly pursued. The appreciation of culture as a barrier was reinforced through a series of activities aimed at ‘reducing resistance’ or ‘convincing’ local populations of taking part in specific interventions. Studies conducted under more culture-centered perspectives included local contexts and community perspectives to inform implementation, as well as specific actions to foster critical thinking in study populations. Considering the different reach and scope of these two approaches is necessary to more realistically inform the impact expected from specific forms of communication formulated under these perspectives.

### Limitations

Defining the boundaries of the communication field has been one of its most important historical challenges [110]. Multiple disciplinary influences involved in the origins of communication studies, as well as the growing fragmentation of what is conceptualized under the communication umbrella, aggravate this situation. Therefore, an important limitation of this review was establishing the characteristics of communication action in interventions that are not reporting specifically on this topic but mention it as a secondary form of intervention. Consequently, search terms might not reflect the full spectrum of communication strategies implemented in LF and CD literature; however, we consider that the terms applied for this review are overarching and depict general trends in the field. Issues of space might have also affected the extent to which communication action was described in included and excluded manuscripts, therefore affecting our analysis. Similarly, we might have failed in identifying implicit definitions of communication, culture and participation included in selected articles. We decided to focus on explicit (textual) references to maintain authors’ perspectives as guide for our analyses. Interviewing authors would be important to further support our claims. In addition, this review was limited to academic literature published in indexed journals. However, communication issues might have been more thoroughly discussed in grey literature and implementation reports. Due to fluency in our research team, this review only included papers in English and Spanish, but we are aware that relevant publications in other languages, particularly French and Portuguese, exist. Finally, three out of the five authors have previous experience working with CD. This might have affected the orientation we took for this analysis.

## Conclusion

As concrete forms of social interaction, health interventions do not happen in a vacuum. NTD elimination efforts exist in contexts historically affected by multiple forms of political and socio-economic marginalization that exacerbate vulnerability to disease. Our results show interventions’ prominent interest on generically promoting community participation or individual behavior change, regardless of the particular conditions of the intervention at hand. Deploying interventions that ignore larger dynamics and that fail to account for the complexity of social life in their design and implementation likely create new forms of silence and neglect. The field of health communication, however, offers theoretical and methodological resources that can help articulate issues of power, representation, and meaning around health issues as expressed in practices at micro and macro levels of interaction, as well as mechanisms that foster dialogues among populations and interventions’ implementers, designers, and researchers.

## Supporting information

S1 Table PRISMA-ScR checklist. (DOCX)

S2 Table Overview of findings identified in lymphatic filariasis’ manuscripts. (DOCX)

S3 Table Overview of findings identified in Chagas disease’s manuscripts. (DOCX)
