# Supplementary material for: Communication in Neglected Tropical Diseases’ elimination: A scoping review and call for action
Source: PLoS Negl Trop Dis. 2022 Oct 13;16(10):e0009774. doi: 10.1371/journal.pntd.0009774 (PMC9595560; doi:10.1371/journal.pntd.0009774)
Supplement: S2 Table — (DOCX) [file pntd.0009774.s002.docx]

| **SI Table 2. Overview of findings identified in lymphatic filariasis’ (LF) manuscripts** | | | | |
| --- | --- | --- | --- | --- |
|  | **Country** | **Authors** | **Approach – Conceptualization and theoretical/ methodological references** | **Implemented strategies - Communication domains and actions** |
| 1 | Ghana | [1] | “Since the mainstay for the control programme is not morbidity control but transmission blockage through chemotherapy, the success of such a strategy depends largely on the level of coverage of the drug administration to the target population. Understanding community perceptions about the disease is vital to encouraging maximum participation.” | **Individual – perception:** “A descriptive survey was carried out to explore perceptions on causes, treatment and prevention of lymphatic filariasis. Perceptions on community participation in disease control programmes were also assessed (…)The study revealed majority of the individuals living in endemic areas not to be aware of how the pathogen gets transmitted. This could be due to lack of adequate information on the cause of the disease among the study communities. It was insightful observing that majority of the respondents identified hydrocoele, one of the clinical signs of lymphatic filariasis as a common health problem. This acknowledgement could be exploited to boost residents’ interest in getting better informed on the disease towards active participation in the control activities, (…) Majority of inhabitants in the study communities lacked the motivation to participate fully in the control programme mainly because they did not see any immediate direct benefit to be derived from the control programme (…) Intensive health educational campaigns in endemic communities are also needed to encourage community participation in the MDA programme.” |
| 2 | India | [2] | “During the camp, Information Education Communication (IEC) materials were used to educate patients on management procedures to be followed up at home.” | **Individual – knowledge**: “LF camps focused on soap and water wash combined with yoga exercises and prescription for care of BEP (…) The investigators developed an IEC booklet of 8 colour pages in local languages. IEC booklets contained information on LF, lymphedema, essential skin care, soap and water wash, dermatology prescriptions for care of BEP, simple yoga exercises and pranayama (special breathing techniques). Booklet contained photos of yoga postures.” |
| 3 | Haiti | [3] | “Policy and discourse in global health continues to stress the importance of community engagement and social mobilization (…) However, this rhetorical normalization hides a more challenging reality: we continue to struggle with the creation of scalable and culturally-competent approaches to engage local people in public health, especially in the social determinants of health (…) Involving local people in environmental sanitation is not only a matter of individual and household behavior change in garbage disposal, water storage, and hygiene. It also involves wider systems of governance and infrastructure that surround larval habitats, and the economic, political, and social contexts that accompany them (…) A large body of applied social science research on infectious disease has shown that better community engagement and behavior change is not only about designing better messages, but more about building trust, social learning, and the ability to iteratively adapt plans based on feedback from community members and field teams [1]. Our approach here assumed that, as Bardosh [36] noted in Uganda, global health interventions are evolving ‘social experiments’. Dominant models and plans require flexibility. Power and politics need to be understood and engaged [37]; and planners and practitioners need to recognize that mistakes need to be made for learning to take place.” | **Community –participation, collective action:**  “Ultimately, through cycles of formal research, feedback, meetings, discussions, brainstorming, and tacit learning, the PKM team developed not only specific education and social engagement strategies but also, perhaps more importantly, the relational capital needed for a viable approach. Community-directed mosquito control, centered on strong local partnerships and participation, developed through various social processes: from our mosquito marker, project pamphlet, written and practical tests to evaluate new potential recruits, repeated community and local leader workshops and meetings, the use of ‘police’ as a metaphor to guide programmatic work, the implementation of our community competitions, and the testing of different management systems (such as sign-in and out sheets) to supervise field workers.” |
| 4 | Haiti | [4] |  | **Interpersonal– diffusion:** “A number of means were used to transfer health education knowledge to the child and his mother. A “hands on” demonstration of proper hand washing techniques and wound care was provided. Pictures and diagrams were used to facilitate the learning process (…) Verbal and written instructions for self-care of the child related to skin care were also provided though professional medical translator who supported the exchange of information from English to Creole during the initial and follow-up NGO sponsored clinics.” |
| 5 | India | [5] | “No doubt, awareness and education play a crucial  role in the elimination of LF” | **Individual - knowledge**: “Intensive behaviour change communication (BCC) activities were carried out to make the community aware of the facts pertaining to LF and cooperate in the consumption of the drug (…) The present study has identified certain aspects in which people need to be sensitized and educated (…) An educational message describing the most frequent, mild side effects along with simple suggestions on how to manage them could alleviate fear and thus increase MDA compliance. The educational message should also indicate that many of the side effects are related to the killing of LF by DEC and that the probability of side effects decreases with the number of microfilaria also decreasing (…) The message that all people living in endemic areas are at risk of infection and that one could be infected even if asymptomatic should be emphasized in upcoming pre-MDA educational campaigns” |
| 6 | Nepal | [6] | “Intensive health education is of utmost importance to raise awareness in people to facilitate an increase towards maximum compliance levels (…) The research suggests if appropriate awareness strategies are targeted at a specific population with an appropriate health education message then it can help to facilitate a change (…) If health education is given to children at a young age, it will have an influence on their attitude and enhance their knowledge and skills [19] (…) Targeting school children who occupy one-third of population could provide a sustainable information flow to larger population as they can act as ‘messengers’ to their family and community.” | **Individual – knowledge:** “Classroom-based interactive health education sessions targeted at school children - educational and interactive components (i.e., group work, drama, quizzes, and games) (…) Intervention manual based on information from existing LF IEC materials (…) Students were asked to develop short key messages and were encouraged to share these messages with their family members and friends.” |
| 7 | Haiti | [7] | “By rejecting a discriminatory approach, PELF acknowledges the right of all batey residents to one specific aspect of the right to health—protection from, and treatment for, LF. This process presupposes that the lives of batey residents are worth reaching, regardless of whether political or legal circles have declared them illegitimate. Recognition of batey residents by PELF contrasts starkly with the loss of recognition accumulated over years of discrimination, structural violence, and the recent Sentencia. In a way, PELF has found itself in the space between the powerful and the weak, between a political and judicial system that considers batey residents as nothing more than cheap laborers, and the residents themselves, whose claims for recognition as people are at stake.” | **Community – collective action:** “Operationally, PELF recognized the need to collaborate with bateyes. Mobilizing communities entailed acknowledging local authority held by neighborhood associations, support systems that seemed to carry more respect among batey residents than the municipal or national government, whose candidates for office were said to make fleeting appearances motivated only by votes. Gaining trust was essential for approaching a population harboring deep skepticism of outsiders, particularly government agencies. Finally, trust and respect could be conveyed in simple interactions between PELF and those they try to reach—such as an unassuming attitude inside a home without a chair. Small gestures indeed, they nonetheless reflect an approach that recasts batey residents as participants in their own health, a rebuttal of the, “state politics of abandonment” that diminish their place in society.” |
| 8 | Tanzania | [8] | “Community mobilization is central. The aim of social mobilization is ‘to stimulate the endemic communities  to own and sustain program interventions and adhere to the strategies being used (…) Community directed distribution has been said to promote a higher uptake of drugs because of its participatory nature, which is assumed to generate a more pronounced sense of ownership and sustainability.” | **Community – participation, structure of program and services:** “The responsibility for advocacy and mobilization is assigned to local government representatives, health workers and community representatives such as community leaders and the selected drug distributors. In Lindi Urban and Lindi Rural, public announcements were made from vehicles. Posters showing people taking drugs were seen in Morogoro Rural, but not elsewhere. In Morogoro Urban, local television and radio stations broadcasted the campaign (…) This study’s data point to a form of participation in which distributors contributed their labour for a minor sum of money to conduct a census, distribute drugs and report on drug uptake among people within a geographically delimited area. The target population was rarely seen to have any active participatory role or decision making power.” |
| 9 | Indonesia | [9] | “The micronarrative survey is based on the recognition that participation with MDA is a social process, rather than a strictly individual one. As such, an individual’s direct and indirect experiences with the MDA and with the people associated with MDA will be most revealing about how the implementation of MDA can be improved. One of the important advantages of working with micronarrative is that it does not constrain the respondent to provide information within a tightly prescribed framework of questions and answer options. Storytelling provides a mechanism to explore both expected and unexpected themes, using the respondent’s personal experience as the reference point for subsequent closed questions.” | **Individual – experience:** “A micronarrative survey tool was developed to capture community members' experience with MDA and the social realm where drug delivery and compliance occur (…)in terms of messaging surrounding the next round of MDA, it was recommended that the district health authorities focus their messages on the social norm of compliance (e.g. “everybody is doing it”) and on the safety of LF drugs globally and in Indonesia. It was suggested that messaging regarding side effects continue to be used, with a focus on promoting the message that side effects indicate that the medicine is working. Ancillary benefits to treatment regarding the elimination of intestinal helminthes should also be promoted, particularly in Agam District. Finally MDA should be promoted as a preventive activity, rather than a treatment (“taking it will keep you healthy”). In order to reach younger persons, use of social media and text messaging were suggested (…) For those who had never complied with taking the LF drugs in the past (…) it was recommended to develop a method to identify these persons at the start of the drug delivery encounter so that the drug distributors could target them with specific messaging (…) In addition, messages about drug safety should be used to help promote trust and reduce fear of side effects for communities (…)“ |
| 10 | Haiti | [10] | “The IEC approach was informed by results of knowledge, attitudes, and practices (KAP) surveys conducted in 2012 and 2013, which asked how people hear about the MDA program. As a result of survey information, a higher proportion of funding was spent on banners, fliers, and radio and television messages, and a lower proportion on posters. Importance was also placed on messaging through megaphones mounted on trucks.” | **Individual – knowledge**: “The information, education and communication (IEC) efforts are multi-channeled, including posters, flyers, banners, sound trucks, radio, television spots, and community meetings held in schools, churches, and markets. Program managers also hold live press conferences on local radio stations, which include answering listener call-in questions (…) For a short period of time, the streets were flooded with volunteers and staff, who were made highly visible by the specially designed NTD program tee-shirts they wore. Messages focused on how to get treatment and on side effects (…) Promoters, including health workers, teachers, and political leaders, are chosen based on having had experience working with the health center in previous health campaigns (e.g., immunization) and having strong standing in the community. As well as recruiting, training, and supervising CDDs and promoters, the community leaders’ role is to sensitize the population at community meetings, schools, churches, and other venues; to meet with and inform other political and church leaders who have influence in the community; and to deliver MDA kits to distribution posts and help manage stock (…) An evidence-based, multi-channel communication approach, combined with high visibility of the MDA itself, resulting in high awareness among the community where the MDA is occurring (…) Focus of key communication messages where to get treatment and on side effects— strong knowledge on disease was not required (…) “ |
| 11 | Fiji | [11] | “We postulate that village structures and roles within a community have a workable attachment to every household that can be explored for mass eradication of LF. Through traditional village forums and following community activities to promote MDA programs, members are able to participate and involve themselves as a way of increasing their awareness about the issue at hand. It also helps them in their drug ingestion choices because they directly access proper information first hand through the forums and then provide the necessary information for participation to their households as a rational decision and not based merely on persuasion.” | **Community – participation:** “Greater adherence to mass drug administration was achieved through the use of village health forums in Fiji to share information about  lymphatic filariasis and by individuals taking roles in community activities regardless of educational attainment (…)Sociocultural understandings of affected community groups are pivotal in achieving sustainability, local participation and ownership. Knowledge and dissemination of information in traditional village forums were better predictors of ingestion of the DEC and ALB in the community.” |
| 12 | India | [12] | “Successful outcome is dependent on operational effectiveness of the MDA programme in terms of interaction of the drug distributor (DD) with the community for social mobilization, outreach and monitoring.” | **Interpersonal - diffusion:** “Interpersonal communication by the DDs at the time of enumeration of the household and during drug distribution is the most important activity in preparing the community for compliance to MDA (…) The electronic and print media were not very effective in propagating the message on MDA (…) Hence, educational and motivational activities need to be planned to cover all the target population.” |
| 13 | India | [13] | “Community-level group participation is found to be a structural aspect of social capital that has a contextual influence on an individual’s health [10] and many studies suggest significant influence of social capital on health behaviours [11–14]. Group-work plays an important role in community education and social support and networking allow groups to work together towards shared goal by  coordinating strategies and pooling resources.” | **Interpersonal – social networks:** “Core approach was formation of small groups in community, strengthening of existing groups and creation of a network of groups for community mobilization. Kudumbasree, a women community network and an initiative of Government of Kerala, exist for every 20 households. Amalgamation of all units in a ward formed an Area Development Society, which was utilized for our intervention activity. Whereever such active groups are lacking, we constituted groups with one family member each from every 50 houses with the help of PHC staff/ ASHA/Anganwadi worker. These groups were thoroughly briefed and they in turn propagated MDA messages in all subsequent weekly meetings. Leaders of all groups of a Health Sub-Centre together formed a networking group and organized discussions were conducted with these groups in all seven intervention sites in Arm I.” |
| 14 | Kenya | [14] | “The elimination campaign is faced with the challenge of persuading people who have no symptoms of the disease to take the drugs and to continue complying for as long as necessary.” | **Individual - knowledge:** “The District Medical staff and politicians were first to be sensitized about MDA (endemicity of the area, purpose of mass treatment, drugs used, method of distribution, length of distribution and role of WHO in the programme) followed by peripheral health staff and community leaders (…) The community leaders then sensitized the community members and together they selected the community drug distributors (CDDs) based on an agreed upon criteria (…) All leaders and stakeholders need to be involved in making the community members understand the importance of taking the drugs and reassuring them that the expected side effects are minor and any severe cases should be reported to the health personnel (…) for improved compliance there is a need to educate and mobilize the community members on all aspects of the Programme well in advance to the distribution time (…) the drug distributors need to be adequately trained and their numbers increased for improved interaction and compliance.” |
| 15 | Kenya | [15] | “Community-based participatory approach is a partnership approach to research that involves community members, organizational representatives, and researchers in aspects of the research process and in which all partners contribute expertise and share decision making and ownership. The aim is to increase knowledge and understanding of a given phenomenon and integrate the knowledge gained with interventions and policy and social change to improve the health and quality of life of community members.” | **Community – participation:** “To elicit more information on opportunities for improved community participation and access to MDA [meetings were held among] stakeholders including governance of health sector, education sector, religious sector, Non-Governmental organization representatives, county commissioner and deputy county commissioners and the local administrative leaders at the wards level, barriers of community participation in the program and opportunities for improved MDA process were identified and intervention measures agreed upon (…) The agenda included presentation of the background and objectives of the study as well as the results of the pre-intervention phase by the Principal Investigator. Minutes of the meetings were taken by one of the study’s co-investigators. The presentation formed a basis of discussion for implementable strategies for improved community participation and access to MDA” |
| 16 | Tanzania | [16] | “It has been argued that social mobilization and advocacy is a key component of effective MDA for lymphatic filariasis in Tanzania (Malecela et al., 2008, p. 117). This is said to comprise everything from radio spots to posters, film shows and the use of mobile megaphones. All this activity is reported to occur in Swahili and to have been incorporated into a set of lymphatic filariasis control ‘programme principles’.” | **Community – participation:** “While these activities [radio spots to posters, film shows and the use of mobile megaphones] may well have occurred at some locations, it is also the case that they either have not occurred or they have had a minimal effect at the field sites where this research occurred. As a result, very few people understood the rationale for distributing drugs, free of charge, to adults and children (…) At all sites there was an eagerness to engage in debate and discussion about these issues, and innumerable complaints that the drugs were being given out by local distributors who could not explain how they worked or exactly what they were for (…) It would be a great mistake to assume that more extensive and sophisticated communication will be sufficient to improve drug uptake. The current system of rolling out free drugs relies upon voluntary labour to run the programme and this, too, is problematic. (…) It may be tempting for policymakers and planners to feel that the most effective way to respond to the low uptake of drugs for lymphatic filariasis is to continue to focus on narrow technical and logistical aspects of drug delivery as there is little they can do to counter rumours that the government and international agencies have hidden agendas that do not serve the interests of local people. This would be a mistake (…) Analysing rumours associated with medical research projects in sub-Saharan Africa, have demonstrated the multiple ways in which stories and rumours articulate local understandings of the workings of power and knowledge (…) Their concerns and questions need to be acknowledged and understood. There is a need for a real engagement with such populations, necessitating different kinds of local-level strategies.” |
| 17 | Zambia | [17] | “Successful undertaking of MDA for LF requires that communities are actively engaged (…) as individuals  may be wary of participating owing to various community level factors(…) Community engagement processes that promote participation are essential to achieving sustainable and successful implementation of MDA for LF (…) They provide an opportunity for improved awareness creation, community empowerment and facilitate programme ownership by the communities.” | **Individual – knowledge and behavior change:** “Community members are administered with an annual dosage of diethylcarbamazine citrate (6 mg/kg) and albendazole (400 mg) by the community drug distributors (CDDs). These are trained community health workers who deliver drugs to the households under supervision of the health facility staff. The CCDs are also responsible for social mobilisation through provision of information, education and communication materials to community members (…) It is pertinent to determine the major local gaps and contexts for development of site-specific strategies. There is a need to emphasize the need for BCC strategy focusing on the safety of drugs and the current importance of MDA. The social group work approach has enhanced the social influence, support and social capital with respect to healthy behaviour adoption for MDA and was successful in enhancing coverage and compliance in Palakkad district.” |
| 18 | Togo | [18] | “The dissemination of the health education message has a two-pronged objective: to identify patients and to educate the general population about the existence of LF as an infectious disease.” | **Individual – knowledge**: “Inform people with a swollen leg that care is available at the local dispensary, through health education, information spread by the administrative system (prefect, village chief, and town crier), and media (posters, radio, newspaper, and television) (…) The use of village volunteers who are also involved in other health programs is an innovative piece of the program, and it is recommended to expand their inclusion in all districts, also the non-LF endemic district. Given cost and time constraints, the training need not be as in-depth of a session, but would at least include the basic mechanisms of disease, simple concepts of treatment, and most importantly that the treatment is inexpensive, effective, and easy to perform. With this information, the village volunteers could effectively function in the roles of message disseminators, case finders and patient recruiters.” |

**References**

1. Aboagye-Antwi F, Kwansa-Bentum B, Dadzie SK, Ahorlu CK, Appawu MA, Gyapong J, et al. Transmission indices and microfilariae prevalence in human population prior to mass drug administration with ivermectin and albendazole in the Gomoa District of Ghana. Parasit Vectors. 2015;8: 562. doi:10.1186/s13071-015-1105-x

2. Aggithaya MG, Narahari SR, Vayalil S, Shefuvan M, Jacob NK, Sushma KV. Self care integrative treatment demonstrated in rural community setting improves health related quality of life of lymphatic filariasis patients in endemic villages. Acta Trop. 2013;126: 198–204. doi:https://doi.org/10.1016/j.actatropica.2013.02.022

3. Bardosh KL, Jean L, Beau De Rochars VM, Lemoine JF, Okech B, Ryan SJ, et al. Polisye Kont Moustik: A Culturally Competent Approach to Larval Source Reduction in the Context of Lymphatic Filariasis and Malaria Elimination in Haiti. Tropical Medicine and Infectious Disease . 2017. doi:10.3390/tropicalmed2030039

4. Byrne SK, Collins SD. Lymphatic Filariasis in Children in Haiti. MCN Am J Matern Nurs. 2015;40. Available: https://journals.lww.com/mcnjournal/Fulltext/2015/07000/Lymphatic_Filariasis_in_Children_in_Haiti.5.aspx

5. Hussain MA, Sitha AK, Swain S, Kadam S, Pati S. Mass drug administration for lymphatic filariasis elimination in a coastal state of India: a study on barriers to coverage and compliance. Infect Dis Poverty. 2014;3: 31. doi:10.1186/2049-9957-3-31

6. Karki P, Prabandari YS, Probandari A, Banjara MR. Feasibility of school-based health education intervention to improve the compliance to mass drug administration for lymphatic Filariasis in Lalitpur district, Nepal: A mixed methods among students, teachers and health program manager. PLoS One. 2018;13: e0203547. Available: https://doi.org/10.1371/journal.pone.0203547

7. Keys H, Gonzales M, Beau de Rochars M, Blount S, Noland GS. Building Trust through Lymphatic Filariasis Elimination: A Platform to Address Social Exclusion and Human Rights in the Dominican Republic. Health Hum Rights. 2018;20: 41–52. Available: https://pubmed.ncbi.nlm.nih.gov/30008551

8. Kisoka W, Mushi D, Meyrowitsch DW, Malecela M, Simonsen PE, Tersbol BP. Dilemmas of community-directed mass drug administration for lymphatic filariasis control: A qualitative study from urban and rural Tanzania. J Biosoc Sci. 2017. doi:10.1017/S0021932016000365

9. Krentel A, Damayanti R, Titaley CR, Suharno N, Bradley M, Lynam T. Improving Coverage and Compliance in Mass Drug Administration for the Elimination of LF in Two ‘Endgame’ Districts in Indonesia Using Micronarrative Surveys. PLoS Negl Trop Dis. 2016;10: e0005027. Available: https://doi.org/10.1371/journal.pntd.0005027

10. Lemoine JF, Desormeaux AM, Monestime F, Fayette CR, Desir L, Direny AN, et al. Controlling Neglected Tropical Diseases (NTDs) in Haiti: Implementation Strategies and Evidence of Their Success. PLoS Negl Trop Dis. 2016;10: e0004954. Available: https://doi.org/10.1371/journal.pntd.0004954

11. Moala-Silatolu A, Nakamura K, Seino K, Kizuki M. Greater Adherence to Mass Drug Administration Against Lymphatic Filariasis through Traditional Village Forums in Fiji. J Rural Med JRM. 2012/11/09. 2012;7: 65–72. doi:10.2185/jrm.7.65

12. Nandha B, Krishnamoorthy K, Jambulingam P. Towards elimination of lymphatic filariasis: social mobilization issues and challenges in mass drug administration with anti-filarial drugs in Tamil Nadu, South India. Health Educ Res. 2013;28: 591–598. doi:10.1093/her/cyt042

13. Nandha B, Meenakshy V, Abdul Khader N, Vijayakumar KN, Jambulingam P. Bridging the gap in outreach and compliance with mass drug administration for lymphatic filariasis elimination in an endemic district in Kerala, India: an intervention research approach. Health Educ Res. 2019;34: 300–309. doi:10.1093/her/cyz005

14. Njomo DW, Amuyunzu-Nyamongo M, Magambo JK, Njenga SM. The Role of Personal Opinions and Experiences in Compliance with Mass Drug Administration for Lymphatic Filariasis Elimination in Kenya. PLoS One. 2012. doi:10.1371/journal.pone.0048395

15. Njomo DW, Kibe LW, Kimani BW, Okoyo C, Omondi WP, Sultani HM. Addressing barriers of community participation and access to mass drug administration for lymphatic filariasis elimination in Coastal Kenya using a participatory approach. PLoS Negl Trop Dis. 2020;14: e0008499. Available: https://doi.org/10.1371/journal.pntd.0008499

16. Parker M, Allen T. Will mass drug administration eliminate lymphatic filariasis? Evidence from northern coastal Tanzania. J Biosoc Sci. 2012/09/27. 2013;45: 517–545. doi:DOI: 10.1017/S0021932012000466

17. Silumbwe A, Halwindi H, Zulu JM. How community engagement strategies shape participation in mass drug administration programmes for lymphatic filariasis: The case of Luangwa District, Zambia. PLoS Negl Trop Dis. 2019;13: e0007861. Available: https://doi.org/10.1371/journal.pntd.0007861

18. Ziperstein J, Dorkenoo M, Datagni M, Drexler N, Murphy M, Sodahlon Y, et al. Final program evaluation methods and results of a National Lymphedema Management Program in Togo, West Africa. J Epidemiol Glob Health. 2013/12/22. 2014;4: 125–133. doi:10.1016/j.jegh.2013.11.001
