# Supplementary material for: Communication in Neglected Tropical Diseases’ elimination: A scoping review and call for action
Source: PLoS Negl Trop Dis. 2022 Oct 13;16(10):e0009774. doi: 10.1371/journal.pntd.0009774 (PMC9595560; doi:10.1371/journal.pntd.0009774)
Supplement: S3 Table — (DOCX) [file pntd.0009774.s003.docx]

| **SI Table 3. Overview of findings identified in Chagas disease’s manuscripts** | | | | |
| --- | --- | --- | --- | --- |
|  | **Country** | **Authors** | **Approach – Conceptualization and theoretical/ methodological references** | **Implemented strategies - Communication domains and actions** |
| 1 | Peru | [1] | “Our analysis was guided by the Health Belief Model, which models health-related behaviours as a function of the perceived threat of the related health condition, perceived benefits and barriers and cues to action (…) The Arequipa campaign stresses the specific objective of convincing community members to accept insecticide spraying; in the community participation literature, this approach is termed as ‘target-oriented’ frame (…) In this context, we adopt a narrow definition of participation: household consent to and completion of insecticide spraying during the first spray of the attack phase.” | **Individual – knowledge:** “In the attack phase, sensibilizadoras (health promoters) visit each household, explain the risks of Chagas disease and the role of the insect in its transmission, and encourage residents to agree to insecticide application. Sensibilizadoras also explain how to prepare the home for insecticide spraying by moving furniture away from walls and stowing bed linens, food and kitchenware.”  **Community – participation:** “Our results suggest that participation in the campaign may have been influenced by neighbour participation. Household participation was clustered spatially, suggesting that neighbour participation may be an important cue to action. While the runs of participation we observed could have been due to spatial patterns of urban vector infestation or other sociodemographic predictors of participation, the positive association between neighbour participation and each household’s participation decision was robust to the addition of vector infestation and neighbourhood type controls in multivariate models. Our results highlight the potential to nudge others towards participation if just one or two households assent to insecticide application. Conversely, one or two refusals on a block may threaten the participation of many additional households.”  **Interpersonal - diffusion:** “We make a second key contribution to the literature on the influence of social norms on health-related behaviour (…) by identifying two important effect modifiers in the relationship between descriptive norms and participation: first, we find that the influence of descriptive norms (ie, observable neighbour participation) is weaker for households with stronger cues to action in the form of visible insect infestation. Second, social influence appears to be stronger in new versus established neighbourhoods. This may be due to the greater importance of social ties for managing risk and transmitting information in poor neighbourhoods.” |
| 2 | Peru | [2] | “Individual behavior change is a critical ingredient in efforts to improve global health. While improving access to high quality health services and strengthening health systems capacity have been important foci of global health and development initiatives, in recent decades the “behavioral revolution” has focused attention on how individual behavior and decision-making drive the effectiveness of most global health programs (…) Finally, the “design for development” movement  has expanded the global health practitioner’s toolkit with new ways to think about innovation, idea generation, user-defined needs, and user experience.” | **Community - participation:** “After several design iterations, three interventions emerged: 1) advanced planning; 2) block leader recruitment; and 3) contingent group lotteries (…) In coordination with the existing MOH campaign, households were approached 7–10 days in advance and asked to commit to participate. Households that agreed to participate were offered convenient 2h appointment windows on their preferred future spray date. A refrigerator magnet with blank spaces to note appointment time and preparation plans was also offered as a planning prompt. In addition, households were given the opportunity to request a phone call or in-person reminder prior to their scheduled appointment. This intervention required additional spray staff to accommodate schedules more tailored and responsive to household requests than in previous campaigns (…) To address low awareness of neighbors’ participation in the campaign and potential distrust of government campaign staff, this intervention recruited formal and informal community leaders to promote participation and persuade reluctant neighbors (…) To address the time and hassle costs of campaign participation, and to encourage communication with neighbors  about the campaign, this intervention used contingent group lotteries to reward households for agreeing to participate in the spray campaign.” |
| 3 | Argentina | [3] | “Text message interventions (TMI) are an important part of the larger strategy of mobile health (mHealth), which entails the utilization of mobile technology in healthcare.” | **Community – patient centered communication:** “Our previous research has shown the interest of pregnant women in Argentina to receive text messaging as a form of communication with healthcare staff (…) According to the information on the admission form, the data collection team sent an SMS reminder to each participant in order to confirm the appointment for the 4-week home visit. Before sending the message the team transferred credit to each phone number so women could reply without having any extra cost. Information related to date and time of the reminder message as well as all replies from participants were transferred to a designed form in order to measure the outcome of interest. Four weeks after delivery, the data collection team performed the scheduled home visits and completed another questionnaire in person to assess if the message had been received and the acceptability of the SMS appointment reminder (…) These data suggest that while urban and rural groups face similar barriers in their access to mHealth, rural populations encounter these barriers more frequently. This is particularly important because one of the often-stated benefits of mHealth is the potential to extend health care and health information to geographically distant or isolated locations.” |
| 4 | USA | [4] | “Citizen science—the engagement of non-scientists in collecting scientific data—has long been used in ecological and wildlife research (…), resulting in an engaged public and providing researchers access to data from large geographic and temporal scales. We pose that citizen science is a powerful yet underutilized tool in public health, given that community engagement is recognized as a core component of many successful public health programs.” | **Individual – knowledge:** “Our program provides resources for people seeking information about Chagas disease and kissing bugs in the U.S., while also requesting kissing bug samples through a variety of media: printed pamphlets phone communication, an educational website solicitations on news stations, and a dedicated email address. The public may submit insect photos through the website or email to be identified by our team, and are invited to submit kissing bug specimens along with associated information.” |
| 5 | Guatemala | [5] | “Participatory action research was used to aid perception and behaviour changes through active stakeholder participation (…) Axial coding was used to generate categories and themes17 based on the Predisposing, Reinforcing, and Enabling Constructs in Educational/ Environmental Diagnosis and Evaluation–Policy, Regulatory, and Organizational Constructs in Educational and Environmental Development (PRECEDE-PROCEED) model.” | **Community – participation, ownership:** “Intervention development included a cyclical process of requesting community members’ input, and engaging them in critical reflection regarding appropriate/ appealing practices, based on the situational analysis of current practices regarding previously identified risk factors (…) The intervention (…) integrated the following: 1) education regarding the disease and risk factors associated with infestation; 2) a modified participatory spraying method including tiled roofs and all walls to eliminate triatomine infestation potentially associated with rodent nests; 3) participatory education and training in mechanical rodent control; 4) participatory education and training in organic waste management combined with productive household activities (i.e. horticulture and chicken rearing); 5) a participant-based reflective process on the intervention.Selected strategies considered participants’ input (…)Participants shared their knowledge, acquired new knowledge, recorded changes they practiced at home, and reflected upon the learning-change process. This approach was intended to build ownership of information and commitment to the learning-change-action effort.” |
| 6 | Argentina | [6] | “Communication difficulties also have a significant impact on the quality of surveillance activities. The lack of timely reporting of reinfestation opens the way for renewed transmission, diminishes the effectiveness of treatment, and increases the risk of generating resistant strains of *T. cruzi*. In our view, the emphasis placed by local health services on one-directional and instrumental communication takes for granted that: a) the health threat is clearly identified by the population at-risk, b) those considered health experts agree on the adopted course of action, c) the at-risk or currently affected population shares a similar degree of alarm to the extent that the message is clear (…) In order for these assumptions to be met, notions and practices of health and disease on both sides of the communication relationship (i.e., the health system/ indigenous population) must be aligned.” | **Community – structure of programs and services:** “Following the mandate of the provincial legal framework, community health workers with indigenous background have gradually joined the provincial health system, improving cultural and geographical accessibility of health care to Aboriginal communities (…)The emphasis on the instrumental aspect of communication – flowing from the health providers towards the Indigenous population – takes for granted that the conditions required for this to occur are met [6]. One condition is that the health threat that Chagas disease represents must obviously be recognized by the at-risk groups (Pilaga and Wichi). This does not occur as even though both groups have rates of infestation and infection that far surpass those prevalent in other groups; their belief systems promote a way of thinking that does not attempt to identify their causes. Another condition is that in order for effective communication to take place, those individuals who take on the role of experts should agree upon the adopted course of action. In the case studied, this was not necessarily true, due to the diversity of therapeutic approaches of both the health personnel and the healers. The third condition is that the at-risk population should have a level of alarm to the extent that the message clearly expresses the nature of the risk. Among the Wichi and Pilaga a reaction of alarm is unlikely due to the naturalization of the presence of the vinchuca in indigenous homes and the absence of an explicit connection between the insect and a disease that is largely silent. The conditions are not met because Western health care providers tend to ignore the beliefs and customs of the target groups, and because the relationships established between the communities and those responsible for health care delivery are conflictive.” |
| 7 | Brazil | [7] | “Several studies have shown that the population has relatively little information regarding Chagas disease (CD) and its vectors; however, this knowledge is relevant because community participation is vital for success in disease control actions.” | **Enabling environment - media:** “Making an educational documentary on CD and its vectors, which could be available to the population for free (…) The documentary was obtained which addresses relevant CD aspects, such as its history, transmission, major vectors and biology, phases of the disease and, in particular, how to inspect the intra-domiciliary and peri-domiciliary areas in search of triatomine bugs or “kissing bugs”. |
| 8 | Argentina | [8] | “La participación comunitaria representa la alternativa más costo-efectiva para el control del vector de la enfermedad de Chagas, incluso, en los entornos con recursos limitados.” | **Individual - knowledge:** “The objective of this study was to evaluate the change in knowledge regarding Chagas disease after the implementation of the strategy of Information, Education and Social Communication component of the local program in the urban area of Avia Terai in 2012 (…) Se realizarón talleres destinados a la comunidad, charlas y diferentes actividades en los establecimientos eductaivos de la localidad dirigidos tanto a alumnos como a docentes y entrevistas en radios locales (…)“After the implementation of a local program of information, education and social communication, an increase in basic knowledge about Chagas disease was observed.” |
| 9 | Ecuador | [9] |  | **Individual - knowledge:** “We delivered a 10-min educational talk about triatomines and Chagas disease at each house (3,191 DUs), supported by a booklet for each family member. The booklet contained color life-size pictures of adults and nymphs of local triatomine species, cartoon- and text-based explanations about triatomine feeding, T. cruzi transmission via feces, drawings of favored triatomine habitats in the intra and peridomicile, games (search the triatomine labyrinth, triatomine color, cut and paste, draw activities and word puzzle), instructions on how to safely collect and report triatomine infestation to the local health promoter, information about Chagas disease signs and symptoms, and practical recommendations to prevent triatomine infestation. A calendar (passive detection device) with educational information was affixed to one of the bedroom walls, usually near the bed. Educational talks, reinforced by presentation of a video, participatory classroom activities, and outdoor didactic games were conducted at the elementary schools in each community.” |
| 10 | Honduras | [10] | “PRECEDE is a framework designed to assess health-related behaviours and environment from epidemiological, social, behavioural, educational, administrative and political perspectives. The subsequent PROCEED stage allows planning, implementation and evaluation of public health interventions based on the assessment (…). We considered the establishment of Chagas disease surveillance systems at health centres as health programme planning, and we hypothesized that the model would facilitate a holistic and systematic analysis for determining key factors at different administrative levels of the Ministry of Health and the community.” | **Community – structure of program and services:** “Training was conducted by the National Chagas Programme (technicians) for the Departmental Health Offices (Epidemiologists and Environmental Health Coordinators), who in turn trained health centre personnel (physicians, nurses and Environmental Health technicians), who trained community health volunteers who oriented the inhabitants. The head of each health centre (physician or nurse) was responsible for integration of vector surveillance into the routine work systems. Responsibilities of health centre staff included; promoting vector search; registering bugs reported by the inhabitants; organizing response with the community health volunteers; and supplying educational materials, spraying equipment and insecticide to the trained community sprayers.” |
| 11 | Guatemala, El Salvador and Honduras | [11] | “To prevent transmission of Chagas disease resulting from re-infestation of houses by T. dimidiata in areas with limited  resources, Guatemala, El Salvador, and Honduras implemented community-based surveillance, in which community members report the presence of bugs in houses to trigger a response by local health services of the Ministry of Health (…) Community-based surveillance has been shown to be effective and cost-effective, but can be challenging to sustain.” | **Community - structure of program and services:** “Community-based vector surveillance for Chagas disease consisted of five essential functions: 1) health promotion—instruction of the community on how to search for bugs; 2) detection of bugs in houses by inhabitants; 3) reporting of bugs to health centers; 4) analysis of reports of bugs, and decision making and planning for response; and 5) response to the report (…) Health center staff and community health volunteers promoted bug searches through different networks including nuclear and extended families, neighborhoods, schools, and churches.” |
| 12 | Bolivia | [12] | “The management of the ecosystem in which domestic vectors develop, with community participation, has long been a promising idea and in some instance has given good results (…) However, changing attitudes and practices in poor indigenous communities which have deeply rooted habits and traditions is a long process. Sustainability is a key parameter in community participation. ‘Good practices’ may be acquired, but if not regarded as a priority could be abandoned in the medium term. In Bolivia where poor farmers do not see Chagas disease as a real health problem, the proposed control techniques of the study may vanish with time.” | **Community - participation:** “The delivery strategy for community participation was identified from the social results and was based on interpersonal communication, community mobilization, lobbying at the community leader level, and supported advertising. The following activities were carried out by the research team: a house-to-house diagnostic of the domestic structures to explain what should be done in each particular situation; a strong social intervention, including school teachers, the participation of community leaders, focus groups, meetings and house-to-house visits (…) Explanations were given to every householder on the correct way to improve their houses with a correct mud wall coating; a technician could help the householder at the beginning if needed, then the householder had to carry on. The intervention phase lasted 6 months and advances were supervised by the research team.” |
| 13 | Guatemala | [13] | “We recommend future interventions focus on long-term control and targeted Ecohealth interventions that limit domestic and peridomestic reinfestation after suppression of the vector by traditional methods. Approaches that combine insecticide use and community-based participatory approaches are expected to offer long-term benefits.” | **Community – participation:** “Interventions also focused on community education regarding Chagas disease and on making houses resistant to reinfestation by *T. dimidiata* through hygienic and targeted home improvements (…) Interventions were performed only upon approval of the head of household and were facilitated by local community leaders who, among other actions, coordinated access to building materials.” |
| 14 | Ecuador | [14] | “We applied a youth participatory research and evaluation (PRE) approach (…) to involve rural and urban youth from Loja Province, Ecuador, in research and evaluation of a Chagas disease prevention program developed in this area (…) Previous research has shown that PRE can help youth to develop their critical and reflexive thinking skills, become more aware of the reality in their communities, enable them to reflect on their own concerns, motivate them to take action in the community, increase their community involvement and enhance their ability to promote community change (…) This approach relies on local knowledge (…) as a way to identify and affirm youth involvement and leadership in the process of discovering, analyzing, and interpreting the changes that unfold in their own social milieu (…) It also emphasizes the epistemological dimension of evaluative processes, conceiving them as privileged opportunities for knowledge production, learning and consciousness- raising.” | **Community – participation:** “A group of adolescents from the communities called the Ñaños (…) was formed by HLI in the summer of 2014 with the purpose of engaging youth in participatory video projects that could help them to document aspects of local development as understood from their own perspective. In 2015, we contacted this group and invited them to take part in a participatory research and evaluation intervention that could help them to implement skills obtained during the first phase of the project, while analyzing HLI’s role in their communities (…) Our findings suggest that youth participatory research and evaluation (PRE) can enhance skills and increase self-esteem and a sense of achievement among youth. It can stimulate knowledge sharing among rural and urban youth and between them and external researchers, and it can facilitate the circulation of grassroots knowledge with regard to the role of youth in their communities.” |
| 15 | Spain | [15] |  | **Individual - knowledge:** “A Chagas disease-specific programme was developed, focused primarily on migrants from Bolivia and Latin American women of childbearing age. Its objectives were to improve migrants’ knowledge and decrease their fears regarding the disease and to encourage them to undergo screening for *T. cruzi* infection, particularly Latin American women of childbearing age. Staff of non-governmental organisations (NGOs) and migrants’ associations promoted talks on the disease to migrants using their services and used a variety of approaches to encourage them to participate, such as placing advertisements on the walls of their premises and talking to people in person or by telephone (…) A culturally tailored leaflet about the disease was designed following the qualitative research (…) From December 2007 to July 2010, we organised talks on Chagas disease to groups of migrants in Madrid, Jerez de la Frontera (Cádiz) and Alicante (…) General information about Chagas disease and information regarding the ongoing programme was also spread through media (press and radio) targeting migrants and social events for people from Latin America, such as the Bolivian National Day celebrations in Madrid in 2008 and 2009.” |
| 16 | Mexico | [16] | “La participación comunitaria, es una estrategia que promueve alianzas entre investigadores y actores sociales (…) podríamos entender a la investigación basada en la participación comunitaria, como un proceso metodológico cualitativo que conjuga los saberes socioculturales con los conocimientos del grupo investigador para mejorar las condiciones de vida de las comunidades.” | **Community - ownership:** “Through [participatory research] tools and with the support of parents, ejidal and school authorities, educational workshops were given to children of the basic education to know the cycle of transmission, and to prevent CD due to the abundance of the vector in the study area. A program called “Small Investigators” was implemented to initiate processes of appropriation and socialization of knowledge in the community.” |
| 17 | Bolivia | [17] |  | **Community – participation:** “Residents carried out the capture of triatomine species according to a strategic methodology based on entomological surveillance with community participation developed by the National Chagas Program (Ministry of Health, Bolivia). Members of each household received an educational folder with instructions on how to search for triatomines in their homes (…) The entomological surveillance folder was designed to be used in tropical non-endemic regions where triatomines are not known to colonize houses. It provides basic information on Chagas disease, a schematic representation of all community players that take part in the initiative, and an entry for family data. Inside there are two figures illustrating both the peridomestic and intradomestic areas with directions on how to clean and maintain each area in order to prevent triatomine colonization. Earlier studies had identified the presence of *Rhodnius robustus* in palm trees of the Tropics of Cochabamba region (…) Thus, the developmental cycle for this species was included in the folder to facilitate vector identification by the participants. Information on how to avoid oral Chagas disease transmission is also provided.” |
| 18 | Argentina | [18] | “La necesidad de pensar la investigación para la producción de recursos de comunicación y educación, desde perspectivas más conscientes y críticas, especialmente si consideramos que con los resultados de nuestros trabajos y la difusión de las herramientas que desde allí surgen, aportamos a la construcción de sentidos y representaciones sobre la compleja trama del Chagas (en la que todos/as estamos implicados/as).” | **Community – meaning making:** “La información recogida permitió construir una aproximación general de las representaciones y vivencias cotidianas que están en la superficie de lo relacional y que determinan procesos sociales de exclusión o inclusión de las personas afectadas por el Chagas. Con fines exploratorios, nos interesaba aproximarnos a cierta información que sirviera de sustento al trabajo de elaboración del guión de un material audiovisual de corta duración (spot) para la divulgación y sensibilización en torno al tema Chagas.”  **Environment – coalition building:** “Los resultados del estudio dejan ver una realidad llena de estigmas, olvido y exclusión para las personas afectadas por el Chagas, situación que se reproduce en contextos endémicos y no endémicos. Por ello, la necesidad tanto de iniciar/sostener una efectiva campaña mundial de información, como de reforzar/multiplicar las acciones específicas de comunicación y educación de todos los actores y sectores implicados (…) El rol que FINDECHAGAS tiene en el aporte y transformación de las maneras de enfrentar la enfermedad y de responder a ella en los contextos en que se encuentran las personas afectadas. Esta es una instancia que promueve y vela porque las personas reciban tratamiento oportuno y acompañamiento adecuado.” |
| 19 | Brazil | [19] | “Measures that call for the promotion of health, which includes improved housing, in addition to solving specific problems, also generate improved well-being, life quality, and individual health (29). Social health determinants improve when there is an intersectoral action; furthermore, participation of the population, health services, and education help sustain these actions and housing programs that do not include participatory procedures should not be promoted.” | **Community - structure of program and services:** “The HIPCDC (…) triggered a process that involves training, household surveys, municipal proposal adjustments, educational workshops, and administrative and technical reorganization, as well as Unified Health System authority integration (…) Integration between the construction sector and the educational project staff in the three municipalities was carried out, with the educational staff helping the workers to contact the local dwellers who would benefit most from the implementation of improvements to their households. The educational staff consisted of FUNASA members, who made speeches to and guided local residents, and who were responsible for training the health workers to continue interacting with the population in terms of the importance of triatomine control in the different municipalities.” |
| 20 | Argentina | [20] | “The primary healthcare model focuses on community participation and social empowerment (…) Broad social participation of multiple sectors may augment the feasibility and sustainability of control interventions, more so in disperse rural areas including various cultural groups (…) Community participation is expected to increase the coverage, effectiveness and sustainability of vector and disease control actions of Chagas and malaria (…) The underlying premise was that participatory methods and multisector cooperation combined with adequate external support would increase diagnosis-and-treatment coverage and adherence relative to historical local levels, manage ADRs effectively and achieve positive therapeutic responses.” | **Community – participation:** “We developed, implemented and tested a strategy to increase access to diagnosis and treatment of human *T. cruzi* infection in sparsely populated rural sections of Pampa del Indio municipality including 13 villages. This strategy, based on strengthened primary healthcare attention and broad social participation, followed an initial phase of intensified vector control and surveillance across the municipality (…) Community workshops were conducted to introduce the research team, communicate the program's objectives and phases, identify local capacities and weaknesses linked to healthcare activities, and establish a permanent channel of communication with local referents (…) Householders of each target village were convened by radio broadcasts and written messages to schoolchildren's parents at each rural school. Participants included local residents, school teachers, healthcare agents, and leaders of social organizations (…) Three meetings with local health personnel (including physicians, technicians, nurses and healthcare agents) were conducted to canvas their experience on attending Chagas disease patients and to identify locally available resources, including diagnostics and logistics (…). Printed leaflets with project information were distributed to consolidate the main messages.” |
| 21 | Argentina | [21] | “Entendemos que una de las riquezas de nuestro trabajo radica en la perspectiva adoptada, al poner en valor no sólo los aportes teóricos metodológicos de disciplinas de las ciencias biológicas y de las ciencias sociales; sino también el intercambio de saberes con los grupos poblacionales, y la participación de esas comunidades, que en estos casos, están sometidas a una misma situación de pobreza y exclusión social” | **Community – participation:** “En cada localidad, convocamos a líderes naturales y representantes institucionales, intentando visualizar y viabilizar -en forma conjunta- estrategias de acción acordes a las características socioculturales de cada comunidad. De este modo, se fueron desarrollando experiencias integradoras, desde una perspectiva de complejidad y atendiendo la singularidad de los grupos poblacionales.” |
| 22 | Honduras | [22] | “The new practices to improve housing can be framed as a preventive innovation, defined as a new idea or behavior that an individual adopts at one point in time in order to lower the probability that some future unwanted event will occur (Rogers 2003). The understanding of the factors associated with the adoption of these new practices and the conditions for their diffusion among community members is key to scaling-up the intervention. According to Rogers (2003), the diffusion of innovation is a process, mediated by social relations, by which an idea or practice, perceived as new by individuals, spreads over time among the members of a community.” | **Community – Participation:** “The project, initiated in two Guatemalan villages, used a participatory approach to promote knowledge of Chagas disease, knowledge sharing, and social and gender equity. The understanding of different gender roles contributed significantly to the design of culturally adapted housing improvement strategies (…) Women, traditionally responsible for keeping up the appearance of the house, were mainly engaged in activities such as filling holes and painting walls with the sand and clay mixture. Men, on the other hand, usually responsible for structural aspects of the home, were engaged in the development of a new flooring method and mostly collected materials, prepared the mixtures, and applied it to floors. Using an Ecohealth approach, it has thus been possible to integrate community participation, conservation of traditional practices, and gender relations in the development of an effective strategy for Chagas disease prevention and control.” |
| 23 | Mexico | [23] | “Ecohealth is based on the use of transdisciplinary participatory research, which may achieve better health outcomes through integrated vector control (… ) Effectiveness and sustainability of vector control relies on community members’ acceptance and adoption of interventions. Community participation in intervention planning, including decisions about logistics and coordination, are likely to result in vector control programs that are better tailored to community members’ needs as well as increased community ownership of the intervention.” | **Community – participation:** “In order to optimize the installation of screens as a barrier against house infestation with triatomines, we engaged multiple stakeholders (community members, local government, social workers/leaders, health center, carpenters, research groups) in a participatory planning and implementation process which was developed from late 2012 to mid-2013. Stakeholders meetings were organized in two of the villages (Teya and Sudzal) to discuss and organize the vector control intervention (…) It was agreed that screens would be installed in the windows of a single room in each house (with two windows on average). This option was also preferred by the research team in order to: 1) respect equity between inhabitants; 2) encourage households to cover additional windows on their own, so that a positive process with a greater active implication and an increased ownership of the intervention by the community could be promoted.” |
| 24 | Mexico | [24] | “A key aspect for the success of these interventions is their acceptance and compliance by the communities and patients, which is in turn affected by their knowledge and perceptions of the disease and the vector.” | **Individual – risk perception/vulnerability:** “Children’s drawings were collected through a drawing contest (…) Primary school students’ aged 6–12 years old from each primary school (six schools in total) from all four villages were invited to participate in the contest (…) Formal instructions describing the contest and its requirements were presented in a pamphlet and a poster to the school officials and teachers. (,,,) Each student was given two weeks to create a drawing on the topic of ‘‘My house and triatomines’’ (‘‘Mi casa y el pic’’, as triatomines are most commonly referred to as a ‘‘pic’’ in Mayan). Children were asked to produce the drawings in their own homes and bring them back to school. The following details were requested in the drawings: 1) To present representative aspects of their community, 2) To include triatomine bugs or ‘‘pic’’ (local Mayan name for triatomines) in their drawing, 3) To show where bugs hide inside/ around the house, and 4) To show what/whom the bugs feed on.” |
| 25 | Guatemala | [25] | “Campaigns are a commonly used approach for public health programs, but the term “campaign” has many different meanings (…) To control Chagas disease, the term “campaign” has been used for massive vector control operations that include insecticide spraying (…) In general, a “campaign” seems to imply an intensive intervention that aims to deliver information, tools or services to a large population in a short time with the expectation that changes in community health-status will occur.” | **Community – community based social marketing** “The central strategy was for inhabitants of the community to perform intensive bug-hunting in their houses. For community mobilisation, various stakeholders, such as the MoH health personnel, community health volunteers, schoolteachers and non-governmental organisation (NGO) workers, were involved in promotional activities (…) The chief of the vector control unit held seven seminars for the intermediate agents to provide the participants with basic information about Chagas disease and the procedures of bug reporting. The vector control unit provided the intermediate agents with a total of 15,000 material packages that contained letter-size promotional flyers, bug reporting forms, lottery tickets and plastic bags for capturing the bugs. The intermediate agents distributed the flyers and plastic bags in their communities with the slogan, Busque la chinche picuda y gane su premio (Look for kissing bugs and win your prize) to inform community inhabitants of the campaign (…) The schoolteachers gave a lesson to schoolchildren and the community leaders or health volunteers visited homes to encourage residents to look for bugs (…) During Chagas Week, community inhabitants searched for bugs in their houses. The community inhabitants were encouraged to capture all suspicious bugs in the plastic bags and to hand the captured bugs to accessible intermediate agents.” |

**References**

1. Buttenheim AM, Paz-Soldan V, Barbu C, Skovira C, Caldern JQ, Riveros LMM, et al. Is participation contagious? Evidence from a household vector control campaign in urban Peru. J Epidemiol Community Health. 2014;68: 103–109. doi:10.1136/jech-2013-202661

2. Buttenheim AM, Levy MZ, Castillo-Neyra R, McGuire M, Toledo Vizcarra AM, Mollesaca Riveros LM, et al. A behavioral design approach to improving a Chagas disease vector control campaign in Peru. BMC Public Health. 2019;19: 1272. doi:10.1186/s12889-019-7525-3

3. Cormick G, Ciganda A, Cafferata ML, Ripple MJ, Sosa-Estani S, Buekens P, et al. Text message interventions for follow up of infants born to mothers positive for Chagas disease in Tucuman, Argentina: a feasibility study. BMC Res Notes. 2015;8: 508. doi:10.1186/s13104-015-1498-9

4. Curtis-Robles R, Wozniak EJ, Auckland LD, Hamer GL, Hamer SA. Combining Public Health Education and Disease Ecology Research: Using Citizen Science to Assess Chagas Disease Entomological Risk in Texas. PLoS Negl Trop Dis. 2015;9: e0004235. Available: https://doi.org/10.1371/journal.pntd.0004235

5. De Urioste-Stone SM, Pennington PM, Pellecer E, Aguilar TM, Samayoa G, Perdomo HD, et al. Development of a community-based intervention for the control of Chagas disease based on peridomestic animal management: An eco-bio-social perspective. Trans R Soc Trop Med Hyg. 2014;109: 159–167. doi:10.1093/trstmh/tru202

6. Dell’Arciprete A, Braunstein J, Touris C, Dinardi G, Llovet I, Sosa-Estani S. Cultural barriers to effective communication between Indigenous communities and health care providers in Northern Argentina: an anthropological contribution to Chagas disease prevention and control. Int J Equity Health. 2014;13: 6. doi:10.1186/1475-9276-13-6

7. Freitas Bianchi T, Velleda dos Santos C, Jeske S, Paula Grala A, Quintana de Moura M, Santos Madia D, et al. Health education in Chagas disease control: Making an educational video. Rev Patol Trop / J Trop Pathol. 2018;47: 116–124. doi:10.5216/rpt.v47i2.54215

8. Genero S, Zorzo LR, Chaparro RM. Impacto de un programa de educación sanitaria sobre los conocimientos básicos de la Enfermedad de Chagas en una población del Noreste argentino. Rev Fac Cienc Med Cordoba. 2018;75: 168–175. doi:10.31053/1853.0605.v75.n4.20004

9. Grijalva MJ, Villacis AG, Ocaña-Mayorga S, Yumiseva CA, Moncayo AL, Baus EG. Comprehensive Survey of Domiciliary Triatomine Species Capable of Transmitting Chagas Disease in Southern Ecuador. PLoS Negl Trop Dis. 2015;9: e0004142. doi:10.1371/journal.pntd.0004142

10. Hashimoto K, Zúniga C, Nakamura J, Hanada K. Integrating an infectious disease programme into the primary health care service: a retrospective analysis of Chagas disease community-based surveillance in Honduras. BMC Health Serv Res. 2015;15: 116. doi:10.1186/s12913-015-0785-4

11. Hashimoto K, Zúniga C, Romero E, Morales Z, Maguire JH. Determinants of health service responsiveness in community-based vector surveillance for Chagas disease in Guatemala, El Salvador, and Honduras. PLoS Negl Trop Dis. 2015;9: e2410. doi:10.1371/journal.pntd.0003974

12. Lardeux F, Depickère S, Aliaga C, Chavez T, Zambrana L. Experimental control of Triatoma infestans in poor rural villages of Bolivia through community participation. Trans R Soc Trop Med Hyg. 2015;109: 150–158. doi:10.1093/trstmh/tru205

13. Lucero DE, Morrissey LA, Rizzo DM, Rodas A, Garnica R, Stevens L, et al. Ecohealth interventions limit triatomine reinfestation following insecticide spraying in La Brea, Guatemala. Am J Trop Med Hyg. 2013;88: 630–637. doi:10.4269/ajtmh.12-0448

14. Marco-Crespo B, Casapulla S, Nieto-Sanchez C, Urrego JGG, Grijalva MJ. Youth participatory research and evaluation to inform a Chagas disease prevention program in Ecuador. Eval Program Plann. 2018;69: 99–108. doi:10.1016/j.evalprogplan.2018.04.009

15. Navarro M, Perez-Ayala A, Guionnet A, Perez-Molina JA, Navaza B, Estévez L, et al. Targeted screening and health education for chagas disease tailored to at-risk migrants in Spain, 2007 to 2010. Eurosurveillance. 2011;16: 19973. doi:10.2807/ese.16.38.19973-en

16. Polanco-Rodríguez AG, Ruiz-Piña HA, Puerto-Manzano FI. La investigación participativa en niños como herramienta en la promoción de la salud para la prevención de la Enfermedad de Chagas en Yucatán, México. Rev BIOMÉDICA; Vol 28, Núm 3 (2017)DO - 1032776/revbiomed.v28i3573 . 2017. Available: https://www.revistabiomedica.mx/index.php/revbiomed/article/view/573

17. Rojas-Cortez M, Pinazo M-J, Garcia L, Arteaga M, Uriona L, Gamboa S, et al. Trypanosoma cruzi-infected Panstrongylus geniculatus and Rhodnius robustus adults invade households in the Tropics of Cochabamba region of Bolivia. Parasit Vectors. 2016;9: 158. doi:10.1186/s13071-016-1445-1

18. Sanmartino M, Saavedra AA, Prat JG i, Barba MCP, Albajar-Viñas P. Que no tengan miedo de nosotros:el Chagas según los propios protagonistas. Interface - Comun Saúde, Educ. 2015;19: 1063–1075. doi:10.1590/1807-57622014.1170

19. Santos CV dos, Bedin C, Wilhelms TS, Villela MM. Assessment of the Housing Improvement Program for Chagas Disease Control in the Northwestern municipalities of Rio Grande do Sul, Brazil . Revista da Sociedade Brasileira de Medicina Tropical . scielo ; 2016. pp. 572–578.

20. Sartor P, Colaianni I, Cardinal MV, Bua J, Freilij H, Gürtler RE. Improving access to Chagas disease diagnosis and etiologic treatment in remote rural communities of the Argentine Chaco through strengthened primary health care and broad social participation. PLoS Negl Trop Dis. 2017;11: e0005336. Available: https://doi.org/10.1371/journal.pntd.0005336

21. Streiger M, Masi R, Mainero MC, del Barco M, Mendocino D, Fabbro D, et al. Perspectiva interdisciplinaria para el abordaje de una enfermedad infecciosa: chagas o tripanosomiasis americana. Rev Salud Pública. 2014;16: 42–47. doi:10.31052/1853.1180.v16.n1.6913

22. Triana DRR, Mertens F, Zúniga CV, Mendoza Y, Nakano EY, Monroy MC. The Role of Gender in Chagas Disease Prevention and Control in Honduras: An Analysis of Communication and Collaboration Networks. Ecohealth. 2016;13: 535–548. doi:10.1007/s10393-016-1141-9

23. Waleckx E, Camara-Mejia J, Ramirez-Sierra MJ, Cruz-Chan V, Rosado-Vallado M, Vazquez-Narvaez S, et al. An innovative ecohealth intervention for Chagas disease vector control in Yucatan, Mexico. Trans R Soc Trop Med Hyg. 2014;109: 143–149. doi:10.1093/trstmh/tru200

24. Yevstigneyeva V, Camara-Mejia J, Dumonteil E. Analysis of Children’s Perception of Triatomine Vectors of Chagas Disease through Drawings: Opportunities for Targeted Health Education. PLoS Negl Trop Dis. 2014;8: e3217. Available: https://doi.org/10.1371/journal.pntd.0003217

25. Yoshioka K. Impact of a community-based bug-hunting campaign on Chagas disease control: a case study in the department of Jalapa, Guatemala. Mem Inst Oswaldo Cruz. 2013;108: 205–211. doi:10.1590/0074-0276108022013013
